# Total evidence phylogeny of Pontederiaceae (Commelinales) sheds light on the necessity of its recircumscription and synopsis of *Pontederia* L.

**DOI:** 10.3897/phytokeys.108.27652

**Published:** 2018-08-29

**Authors:** Marco O. O. Pellegrini, Charles N. Horn, Rafael F. Almeida

**Affiliations:** 1 Universidade de São Paulo, Departamento de Botânica, Rua do Matão 277, CEP 05508-900, São Paulo, SP, Brazil Universidade de São Paulo São Paulo Brazil; 2 Newberry College, Department of Sciences and Mathematics, 2100 College Street, Newberry, SC 29108, USA Newberry College Newberry United States of America; 3 Universidade Federal de Minas Gerais, Programa de Pós-Graduação em Biologia Vegetal, Avenida Antonio Carlos 6627, CEP 31270-901, Belo Horizonte, MG, Brazil Universidade Federal de Minas Gerais Feira de Santana Brazil

**Keywords:** Aquatic flora, *
Eichhornia
*, *
Monochoria
*, pickerelweed, *
Reussia
*, water-hyacinth

## Abstract

A total evidence phylogeny for Pontederiaceae is herein presented based on new morphological and previously published molecular data. Our results led us to re-circumscribe *Pontederia* to include *Monochoria*, *Pontederias.s.* and the polyphyletic *Eichhornia*. We provide the needed ten new combinations and 16 typifications, arrange a total of 25 accepted species (six representing re-established names) in 5 new subgenera. Furthermore, we provide an identification key for the two genera accepted by us in Pontederiaceae, an identification key to the subgenera, identification keys to the species of each subgenus and commentaries on *Pontederias.l.*, as well as for each subgenus and each species.

## Introduction

Pontederiaceae is a small aquatic monocot family, placed in Commelinales as sister to Haemodoraceae, with both families being sister to Philydraceae (Saarela et al. 2008). This clade can be morphologically characterised by its: distichously-alternate and unifacial leaves, with xylem and phloem alternate (or rarely phloem circular with central xylem); the presence of styloid crystals; perianth whorls partially to completely connate forming a hypanthium, perianth petaloid, flowers bisexual, zygomorphic and enantiostylous; pollen shed with raphides; the presence of placental sclereid idioblasts; and seeds longer than wide with longitudinal wings or striations (Simpson 1990; Prychid et al. 2003; Simpson and Burton 2006; Pellegrini, unpublished data). Furthermore, the relationship between Pontederiaceae and Haemodoraceae is morphologically supported by their endothecium with a basal thickening, non-columellate-tectate exine and the presence of septal nectaries (Simpson 1987, 1990). Pontederiaceae can be easily distinguished from the remaining families of Commelinales by its roots not sand-binding; dimorphic, late bifacial and ligulate leaves, ptyxis involute enclosing the petiole of the preceding leaf; xylem and phloem alternate near the centre of the blades, plus xylem abaxial and phloem adaxial near the margins; bisulcate pollen grains; and the presence of an anthocarp (Arber 1925; Simpson 1987, 1990; this study). The family is currently arranged in four genera (i.e. *Eichhornia* Kunth, *Heteranthera* Ruiz & Pavón, *Monochoria* C.Presl and *Pontederia* L.) and possesses ca. 45 species (Lowden 1973; Horn 1985; Cook 1989; Pellegrini 2017a; Pellegrini and Horn 2017). Pontederiaceae has a pantropical distribution, with the Neotropical region as its diversity centre, where ca. 70% of its species can be found (Barrett 2004; Pellegrini and Horn 2017). Furthermore, Brazil retains most of the diversity for the group, with 24 species known to occur in all kinds of aquatic and damp environments (BFG 2015; Pellegrini and Horn 2017). Despite being unquestionably monophyletic (Eckenwalder and Barrett 1986; Graham and Barrett 1995; Kohn et al. 1996; Barrett and Graham 1997; Graham et al. 1998, 2002; Ness et al. 2011), generic boundaries in Pontederiaceae are still in great need of revision (Ness et al. 2011; Pellegrini 2017a). A total of 30 genera have been described and assigned to Pontederiaceae throughout the years (eMonocot 2010; Govaerts 2018; Tropicos.org 2018) and some authors have accepted up to nine genera in the family (e.g. Cook 1998). All phylogenetic studies invariably recover most genera as non-monophyletic, with *Eichhornia* and *Heteranthera* being the most problematic groups (Eckenwalder and Barrett 1986; Graham and Barrett 1995; Kohn et al. 1996; Barrett and Graham 1997; Graham et al. 1998, 2002; Ness et al. 2011). Based on these published phylogenies, it is clear that these genera have been circumscribed based either on autapomorphic or homoplastic characters. Thus, traditionally proposed generic boundaries need to be urgently revisited.

Recently, *Heteranthera* was recircumscribed to include *Hydrothrix* Hook.f. and *Scholleropsis* H.Perrier, thus being finally rendered monophyletic (Pellegrini 2017a). Nonetheless, the *Pontederia* clade (i.e. *Eichhornias.l.*, *Monochoria* and *Pontederia*) remains neglected (Pellegrini 2017a), with the hopelessly polyphyletic *Eichhornia* being recovered as three distinct lineages within it (Eckenwalder and Barrett 1986; Graham and Barrett 1995; Kohn et al. 1996; Barrett and Graham 1997; Graham et al. 1998, 2002; Ness et al. 2011). The first *Eichhornia* lineage is composed by the erect-emergent, non-clonal species, with perianth spirally-coiled at post-anthesis. The second lineage is composed exclusively by *E.crassipes* (Mart.) Solms, which is characterised by its free-floating and stoloniferous rosette, flabellate ligules and its peculiarly inflated petioles. The last *Eichhornia* lineage is composed by procumbent-emergent species, with distichously-alternate leaves evenly distributed along the stems, infundibuliform perianth and glabrous styles (Pellegrini and Horn, pers. observ.).

According to Pellegrini (2017a), there are two approaches for solving the generic limits in the *Pontederia* clade: (1) sink *Eichhornia* and *Monochoria* into a broader, but morphologically cohesive *Pontederia*; or (2) split *Eichhornia* into three ill-defined genera, in order to maintain *Pontederia* and *Monochoria* as independent genera. The first option is considerably more taxonomically stable and would greatly facilitate the identification of Pontederiaceae specimens, especially for the non-specialists, ecologists, plant growers, farmers etc.

Here, we present a total evidence phylogeny for Pontederiaceae, based on plastid and morphological data, in order to recircumscribe *Pontederia* to include *Eichhornia* and *Monochoria* and provide an identification key to the genera in Pontederiaceae. We also present a synopsis for *Pontederias.l.*, with an updated description for the genus, propose five new subgenera, provide an identification key to the accepted subgenera of *Pontederia* and provide identification keys to the species of each subgenus. Finally, we propose the needed 10 new combinations, present six new synonyms and accept a total of 25 species, five of these representing reestablished names. The present study concludes the bi-generic classification of Pontederiaceae initiated by Pellegrini (2017a) and is a result of the first author’s ongoing systematic studies on Commelinales.

## Methods

### Taxonomy

Specimens from the following herbaria were analysed: AAU, ALCB, B, BA, BAF, BHCB, BHZB, BLH, BM, BOL, BOTU, BR, BRIT, C, CAS, CEPEC, CESJ, COL, CORD, CTES, CVRD, DS, E, EA, ESA, F, FCAB, FLOR, FURB, G, GH, GMUF, GOET, GUA, HAL, HAMAB, HAS, HB, HBR, HERBAM, HNMN, HRB, HRCB, HSTM, HUEFS, HUFSJ, HURB, IAC, IBE, ICN, INPA, IPA, K, KANU, L, LE, LG, LIL, LL, LP, M, MA, MBM, MBML, MG, MO, MVM, MY, NBYC, NY, OS, P, PH, PMSP, PR, PRC, PRE, R, RB, RFA, RFFP, S, SMU, SP, SPF, SRGH, TEX, UEC, UMO, UNA, UPCB, US, USF, VDB, VIC, W and WAG (herbaria acronyms according to Thiers, cont. updated). Fresh specimens, field notes, photographs and specimens for cultivation were gathered by the authors during several field trips across North, Central and South America, between 1980 and 2017. The indumentum and shape terminology follow Radford et al. (1974); the inflorescence terminology and morphology follow Weberling (1965, 1989) and Panigo et al. (2011), as implemented by Pellegrini and Horn (2017); fruit terminology follows Spjut (1994); and seed terminology follows Faden (1991). Species distribution is based on literature, herbarium specimens and fieldwork data.

### Morphological character selection, coding, mapping and morphological analysis

Characters were scored mainly from living specimens in the field and specimens in cultivation and later complemented by spirit and herbarium samples from the aforementioned herbaria. When no living or herborised specimens were available for examination, information was taken from published literature. We have studied at least five specimens for each taxon, with the most representative specimen chosen as the voucher for the morphological matrix (Table 1). Some characters were chosen based on previous studies (i.e. Eckenwalder and Barrett 1986; Simpson 1987; Barrett and Graham 1997; Simpson and Burton 2006), with most characters being scored for the present study. Character coding followed the recommendations of Sereno (2007) for morphological phylogenies. Primary homology hypotheses (De Pinna 1991) were proposed for root, stem, leaf, inflorescence architecture, floral, fruit, seed, palynological and anatomical characters. A total of 96 discrete micro- and macromorphological characters were scored, being treated as unordered and equally weighted (Suppl. material 1).

**Table 1.** Voucher specimens used in the morphological and combined phylogenetic analyses, and Genbank accession numbers for all DNA regions sampled in this study. ^*^Type species of the genus.

Family	Species	Collector & no.	Herbarium acronym	ndhF	rbcL
Philydraceae	*Helmholtziaacorifolia F.Muell.	Mueller 1876	K	EF422989.1	AF206774.1
Philydraceae	*Philydrumlanuginosum Banks & Sol. *ex* Gaertn.	Banks & Solander s.n.	BM barcode BM000990702	U41622.2	U41596.2
Haemodoraceae	*Anigozanthosflavidus* DC.	Brown s.n.	K barcode K000846259	EF422987.1	EF422992.1
Haemodoraceae	*Xiphidiumcaeruleum Aubl.	Perdiz 2376	RB	AF547013.1	AY149359.1
Pontederiaceae	*Monochoriacyanea* (F.Muell.) F.Muell.	Leichhardt s.n.	K barcode K000873493	U41613.1	U41588.1
Pontederiaceae	*Monochoriakorsakovii* Regel & Maack	Maack s.n.	K barcode K000873544	U41615.2	U41590.1
Pontederiaceae	*Monochoriahastata (L.) Solms	Hermann s.n.	BM barcode BM000621681	U41614.1	U41589.1
Pontederiaceae	*Monochoriavaginalis* Burm.f.	Boeea 8471	US	U41616.1	KX527476.1
Pontederiaceae	*Eichhorniacrassipes* (Mart.) Solms	Martius 60	M	FJ861142.1/ U41599.2	FJ861142.1/ EF422991.1
Pontederiaceae	*Eichhorniadiversifolia* (Vahl) Urb.	Harley 10248	RB	U41600.1	U41575.1
Pontederiaceae	*Eichhorniaazurea (Sw.) Kunth	Martinelli 18669	RB	U41598.1	U41573.1
Pontederiaceae	*Eichhorniaheterosperma* Alexander	Smith 2290	NY	U41601.1	U41576.1
Pontederiaceae	*Eichhorniapaniculata* (Spreng.) Solms	Machado 574	RB	U41603.1	U41578.1
Pontederiaceae	*Eichhorniaparadoxa* (Mart.) Solms	Harley 21401	K	U41607.1	U41579.1
Pontederiaceae	*Pontederiacordata L.	Barton s.n.	PH barcode PH00038346	U41617.1	U41592.1
Pontederiaceae	*Pontederialancifolia* Muhl.	Muhlenberg 242	PH	U41618.1	U41593.1
Pontederiaceae	*Pontederiarotundifolia* L.f.	Alvarenga 952	RB	U41620.1	U41595.1
Pontederiaceae	*Pontederiaovalis* Mart.	Pellegrini 474	RB	U41619.1	U41594.1
Pontederiaceae	*Pontederiasagittata* C.Presl	Catharino 342	RB	U41621.1	U41597.1
Pontederiaceae	*Heterantheragardneri* (Hook.f.) M.Pell.	Gardner 1863	K	U41606.2	U41582.1
Pontederiaceae	*Heterantherarotundifolia* (Kunth) Griseb.	Walter 6644	RB	U41610.1	U41585.1
Pontederiaceae	*Heterantheralimosa* (Sw.) Willd.	Assunção 721	RB	U41608.2	U41583.1
Pontederiaceae	*Heterantherazosterifolia* Mart.	Fontana 8316	RB	U41612.1	U41587.1
Pontederiaceae	*Heterantheraseubertiana* Solms	Gardner 1864	BM	U41611.1	U41586.1
Pontederiaceae	*Heterantheraoblongifolia* Mart. *ex* Schult. & Schult.f.	Araújo 38	RB	U41609.1	U41584.1

Data were entered into a matrix of characters per taxa using the software Mesquite 3.20 (Maddison and Maddison 2017; Suppl. material 2). All characters were treated as unweighted and unordered. Maximum Parsimony (MP) analysis was performed using PAUP* 4 (Swofford 2003), with a heuristic search with 1000 random taxon additions and tree bisection-reconnection (TBR) branch swapping. Consistency index (CI) and retention index (RI) were used to assess the degree of homoplasy in the dataset and ACCTRAN (accelerated transformation optimisation; Swofford and Maddison 1987) was used for character optimisation. Statistical support for each branch of the cladogram was evaluated with Bootstrap Support (BS) analyses with 1000 random addition replication. The search parameters used to estimate the bootstrap values were the same as the initial heuristic search. The Bremer Index (BI) was also used to evaluate clade reliability based on the presence of secondary homologies (Bremer 1994). The Bremer Index was calculated by increasing the number of the optimal tree steps until all clades collapsed. Mesquite 3.20 was used to reconstruct the ancestral character states, while WinClada ver. 1.0000 (Nixon 2002) was used to trace the synapomorphic characters on the strict consensus tree.

### Taxon sampling, alignment and phylogenetic analysis

Sequences of the genes *ndhF* and *rbcL* were retrieved from GenBank for 26 taxa representing all currently accepted genera in Pontederiaceae, including outgroups *Anigozanthos* Labill. and *Xiphidium* Aubl. (Haemodoraceae) and the tree was rooted with Philydraceae. All sequences were aligned using Muscle (Edgar 2004) implemented on Geneious software (Kearse et al. 2012), with subsequent adjustments in the preliminary matrices made by eye.

Combined analyses of the plastid regions and plastid+morphology datasets were performed. Prior to combining our data, we performed the incongruence length difference (ILD) test (Farris et al. 1994) to investigate the incongruence between DNA data sets. Analyses, using maximum parsimony (MP) on both matrices, were conducted with PAUP* 4 (Swofford 2003). A heuristic search was performed using TBR swapping (tree-bisection reconnection) and 1000 random taxon-addition sequence replicates with TBR swapping limited to 15 trees per replicate in order to prevent extensive searches (swapping) in suboptimal islands, followed by TBR in the resulting trees with a limit of 1000 trees. In all analyses, the characters were equally weighted and unordered (Fitch 1971). Relative support for individual nodes was assessed using non-parametric bootstrapping (Felsenstein 1985), with 1000 bootstrap pseudo-replicates, TBR swapping, simple taxon addition and a limit of 15 trees per replicate.

For the DNA partitions of the model-based approach, we selected the model using hierarchical likelihood ratio tests (HLRT) on J Modeltest 2 (Darriba et al. 2012. For the morphological partition, the standard discrete Markov model (Mkv) was used, following Lewis (2001) with rates set to equal. A Bayesian analysis (BA) was conducted with mixed models and unlinked parameters, using MrBayes 3.1.2 (Ronquist and Huelsenbeck 2003). The Markov Chain Monte Carlo (MCMC) was performed using two simultaneous independent runs with four chains each (one cold and three heated), saving one tree every 1000 generations, for a total of ten million generations. We excluded as ‘burn in’ trees from the first two million generations and tree distributions were checked for a stationary phase of likelihood. The posterior probabilities (PP) of clades were based on the majority-rule consensus, using the remaining trees, calculated with MrBayes 3.1.2 (Ronquist and Huelsenbeck 2003).

## Results

### Morphological analysis

**Figure 1.**
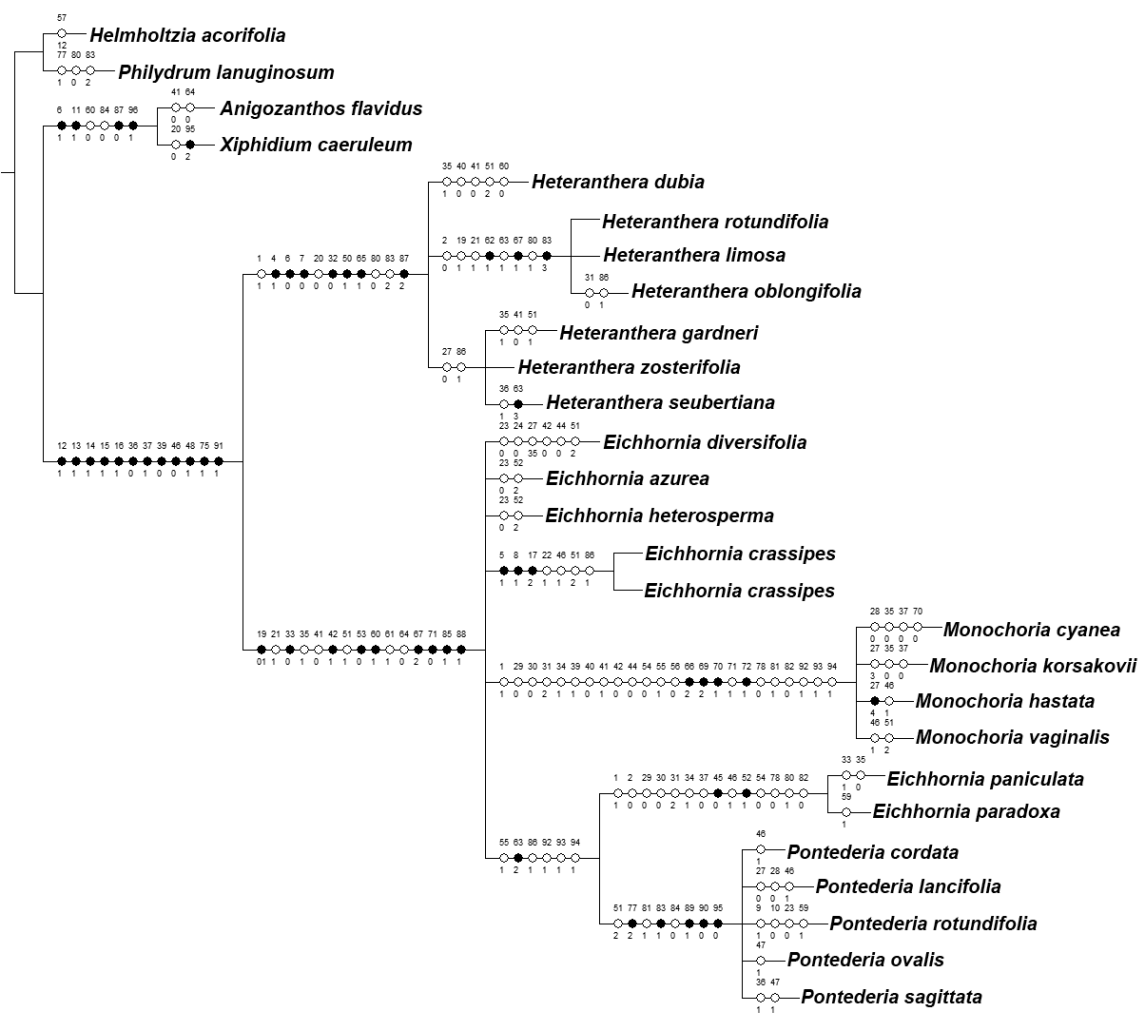
Strict consensus tree (length=209 steps; CI=0.5913; RI=0.8618) recovered by the morphological dataset, showing the character state optimisations at each node of the cladogram, represented by circles. In each circle, the numbers above and below represent the character and character state numbers, respectively (as presented in Suppl. material 1).

**Figure 2.**
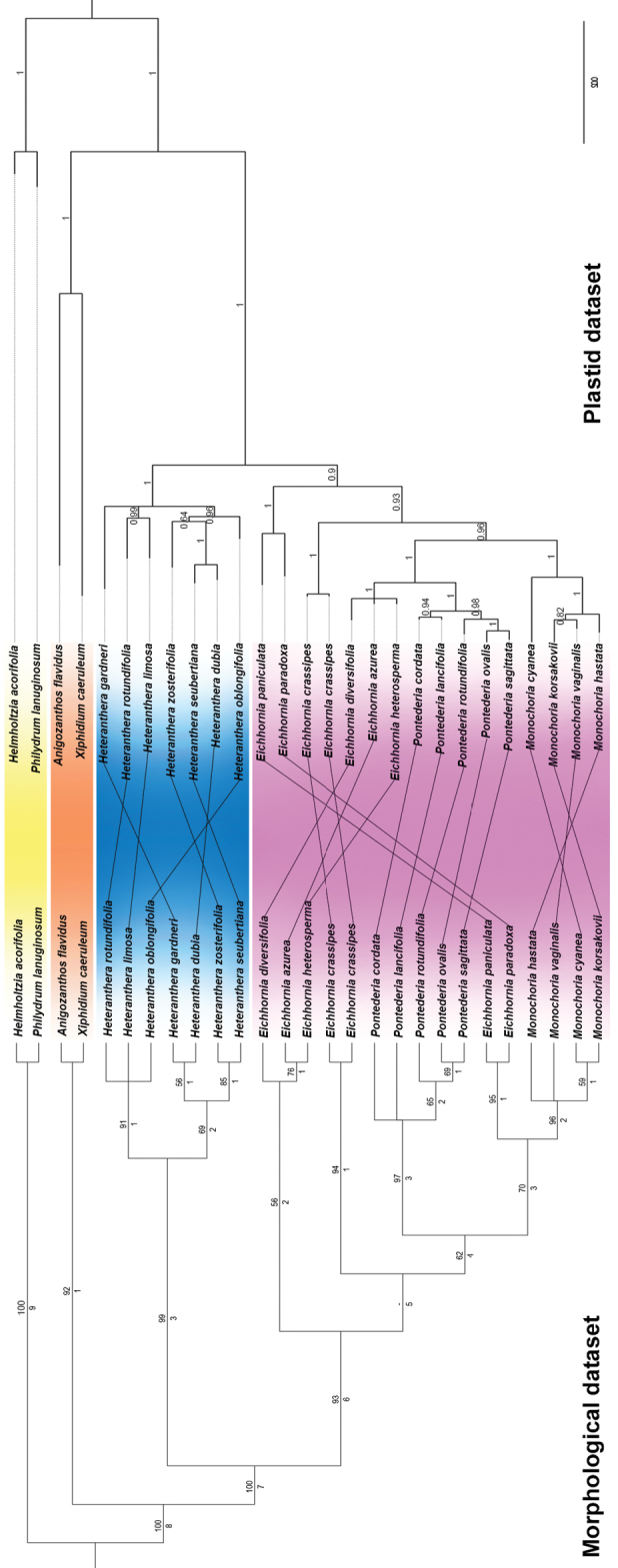
Majority-rule tree recovered for the morphological and plastid datasets. Morphology: bootstrap support values are depicted over the branches, while Bremer Index support values are depicted under the branches. Plastid: posterior probability values are depicted over the branches. Yellow: Philydraceae. Orange: Haemodoraceae. Blue: *Heterantheras.l.* Pink: *Pontederias.l.*

The cladistic analysis retrieved 228 equally parsimonious trees with 209 steps, Consistency Index (CI) of 0.5913, Homoplasy Index (HI) of 0.4087, Retention Index (RI) of 0.8618 and Rescaled Consistency Index (RC) of 0.5096. All 96 coded characters were parsimony-informative. The strict consensus (Fig. 1) and the majority-rule trees (Fig. 2) are presented and discussed below.

The Haemodoraceae+Pontederiaceae clade is supported by seven characters: the presence of septal nectaries (Character 44), perianth 6-lobed (Character 58, plesiomorphic), perianth with 3+3 arrangement (Character 59, plesiomorphic), epipetalous stamens (Character 66, homoplastic), stamens dimorphic (Character 69), endothecium with a basal thickening (Character 72) and non-tectate-columellate exine (Character 76). Pontederiaceae is recovered as monophyletic with high statistical support (BS=100; BI=7; Fig. 2), being supported by: dimorphic leaves (Character 12), leaf-blades late bifacial (Character 13), involute ptyxis where the blade of the new leaf encloses the petiole of the preceding leaf (Character 14), leaf-blades with xylem and phloem alternate in the central portion of the blade and xylem abaxial and phloem adaxial at the margins (Character 15), the presence of a ligule (Character 16), non-equitant leaves (Character 18, reversion), sessile leaves early-deciduous (Character 18), inflorescence deflexed at post-anthesis and in fruit (Character 37), sessile flowers (Character 39), absence of fibrillar tannin cells in the perianth (Character 47), presence of aerenchymatous tissue in the receptacle (Character 48) and in the perianth (Character 49), perianth connate producing a conspicuous tube (Character 56, homoplastic), perianth ranging from lilac to purple or blue (Character 57, homoplastic), posterior lobe(s) with a nectar guide (Character 63, homoplastic), pollen grains bisulcate (Character 75), presence of aerenchymatous tissue in the ovary walls (Character 79) and the presence of an anthocarp (Character 91).

*Heterantherasensu*Pellegrini (2017a), is recovered as monophyletic with high statistical support (BS=99; BI=3; Fig. 2). It is supported by: plants mostly to completely submersed (Character 3, homoplastic), indefinite base (Character 4), water-binding/mucilaginous roots (Character 6), rhizome absent (Character 7), stems freely branching and elongated (Character 9 and 10, homoplastic), ligules 2–several parted (Character 17), spirally-alternate sessile leaves (Character 18), sessile leaves evenly distributed along the stem (Character 20, homoplastic), basal bract conduplicate (Character 30), main florescence reduced to a solitary cincinnus (Character 32), sparse aerenchymatous tissue in the perianth (Character 49), perianth tubular (Character 50), filaments obliquely inserted (Character 65) and unevenly trilobate stigma (Character 87). Within *Heterantheras.l.*, we recover two main clades in the majority rule (Fig. 2), with only one of these being also recovered in the strict consensus (Fig. 1). The *H.limosa* group is composed by *H.limosa* (Sw.) Willd., *H.oblongifolia* Mart. *ex* Schult. & Schult.f. and *H.rotundifolia* (Kunth) Griseb., being characterised by: the absence of clonal reproduction (Character 2, homoplastic), sessile leaves late-deciduous (Character 19, homoplastic), petiolate leaves with elliptic to ovate blades (Character 27, homoplastic), the posterior perianth lobe with flanged base (Character 62) and a nectar guide consisting of a sole spot or dark band (Character 63, homoplastic), sigmoid filaments (Character 67), ovary hemiseptalous (Character 80, homoplastic), axile-parietal placentation (Character 83) and placentation 2-flanged (Character 84, homoplastic). The second clade, named by us as the *H.dubia* group, is composed of *H.dubia* (Jacq.) MacMill., *H.gardneri* (Hook.f.) M.Pell., *H.seubertiana* Solms and *H.zosterifolia* Mart. This group is characterised by: the presence of cleistogamous flowers (Character 43), inflated filaments (Character 68), gynoecium 1-locular (Character 77, homoplastic), ovary aposeptalous (Character 80, homoplastic), intrusive-parietal placentation (Character 83, homoplastic) and placentation slightly 2-flanged (Character 84).

*Pontederias.l.* is also recovered as monophyletic with high statistical support (BS=93; BI=6; Fig. 2), being supported by: distichously-alternate sessile leaves (Character 18), petiolate leaves pulvinate (Character 25), tristylous flowers (Character 42), dense aerenchymatous tissue in the perianth (Character 49), perianth campanulate or infundibuliform or hypocrateriform (Character 50, homoplastic), perianth coiled and tightly enclosing the fruit at post-anthesis (Characters 53 and 55), perianth lobes equal in shape in the same whorl (Character 60) and with obtuse apex (Character 61, homoplastic), stamens 6 (Character 64, reversion), filaments J-shaped or recurved-decurved (Character 67), anthers dorsifixed (Character 71), style J-shaped (Character 85), stigmas evenly trilobate to trifid or capitate (Characters 87), stigma wet (Characters 88), anthocarp tightly enveloping the fruit (Character 92) and anthocarp hardened and ornamented (Characters 93 and 94). *Pontederias.l.* is recovered by us arranged in five clades in the strict consensus (Fig. 1) and in the majority rule (Fig. 2). The *E.paniculata* group is highly supported (BS=95; BI=1; Fig. 2), being composed by *E.paniculata* (Spreng.) Solms and *E.paradoxa* (Mart. *ex* Schult. & Schult.f.) Solms. It is characterised by: its annual life cycle (Character 1, homoplastic), the lack of clonal reproduction (Character 2, homoplastic), inflated sheath of the leaf subtending the inflorescence (Character 29, homoplastic), flat basal bract (Character 30, homoplastic) with a caudate apex (Character 31, homoplastic), main florescence with a fistulose main axis (Character 34, homoplastic), inflorescence erect at post-anthesis and in fruit (Character 37, reversion), floral organs lacking tannin cells of the homogeneous type (Character 45), perianth with a moderate amount of granular tannin cells (Characters 51 and 52), perianth spirally-coiled at post-anthesis (Character 54, homoplastic), ovary walls lacking tannin cells (Character 78, homoplastic), ovary hemiseptalous (Character 80, homoplastic) and septae lacking tannin cells (Character 82, homoplastic). Based on morphology, *E.meyeri* A.G.Schulz should also be placed in the *E.paniculata* group. *Monochoria* is recovered as monophyletic with high statistical support (BS=96; BI=2; Fig. 2), being characterised by eight non-homoplastic synapomorphies: pedicellate flowers (Character 39, reversion), perianth only basally connate (Character 56, reversion), absence of a nectar guide (Character 63, reversion), presence of a petalo-staminal tube (Character 66), stamens unequal (Character 69), presence of a filament appendage (Character 70), enantiostylous flowers (Character 71, reversion) and poricidal anthers (Character 72). *Eichhorniacrassipes* is recovered as a sole species with high statistical support (BS=94; BI=1; Fig. 2), being characterised by: its free-floating habit (Character 5), the production of new rosette through stolons (Character 8), flabellate ligules (Character 17), spirally-alternate petiolate leaves (Character 22, homoplastic), perianth loosely enveloping the fruit (Character 55, homoplastic) and nectar guide consisting of a sole spot (Character 63, homoplastic). *Eichhornias.s.* was recovered with low statistical support (BS=56; BI=2; Fig. 2), being composed by *E.azurea* (Sw.) Kunth, *E.diversifolia* (Vahl) Urb. and *E.heterosperma* Alexander. It is characterised by: growing as mostly submerged plants (Character 3, homoplastic), stems freely branching and elongated (Character 9 and 10, homoplastic), sessile leaves late-deciduous (Character 19, homoplastic), petiolate leaves evenly distributed along the stem (Character 23, homoplastic), flowers self-compatible (Character 38, homoplastic), floral tissues lacking granular tannin cells (Character 46, homoplastic) and presenting fibrillar tannin cells (Character 47, reversion), nectar guide consisting of a sole spot or dark band (Character 63, homoplastic) and ovary walls lacking aerenchymatous tissue (Character 79, reversion). Finally, *Pontederiasensu*Lowden (1973) was recovered by us as monophyletic with high statistical support (BS=97; BI=3; Fig. 2). It is characterised by: flowers self-compatible (Character 38, homoplastic), nectar guide consisting of two spots (Character 63, homoplastic), pseudomonomerous ovary (Character 77), the presence of epithelial cells in the septae (Character 81, homoplastic), pendulous and unflanged placentation (Characters 83 and 84), fruit an achene (Character 89), seeds one per locule (Character 90) and smooth testa (Character 95). Nonetheless, the subgenera proposed by Lowden (1973) cannot be maintained, due to *P.rotundifolia* L.f. (i.e. P.subg.Reussia) being nested within P.subg.Pontederia (*sensu*Lowden 1973).

### Plastid and combined analyses

The *ndhF* characters represented 503 characters of the plastid dataset, with GTR+G as the nucleotide model selected. The *rbcL* characters represented 1355 characters of the plastid dataset, with HKY+G+I as the nucleotide model selected. The plastid dataset represented 1858 characters, of which 241 characters were variable and 119 characters were parsimony-informative. The plastid Bayesian analysis recovered a mostly resolved tree with 23 well-supported clades (>PP 95%) (Fig. 2). The congruence between the plastid and morphological datasets is illustrated in Figure 2. In both analyses, *Pontederias.l.* and *Heterantherasensu*Pellegrini (2017a) are strongly supported, but the relationship between the species is greatly different. In *Heteranthera*, the morphologically based topology is better resolved and recovers two clades, while the plastid dataset recovers two clades plus *H.gardneri* in a polytomy (Fig. 2). In *Pontederias.l.*, both datasets recover the genus arranged in five clades, but the relationship between them is different. In the morphological dataset, *Eichhornias.s.* is the first lineage to diverge, followed by *E.crassipes*, *Pontederias.s.* and *Monochoria*, sister to the *E.paniculata* group. Alternatively, in the plastid dataset, the *E.paniculata* group is undoubtedly recovered as the first lineage, followed by *E.crassipes*, *Monochoria* and *Pontederias.s.*, sister to *Eichhornias.s.*

**Figure 3.**
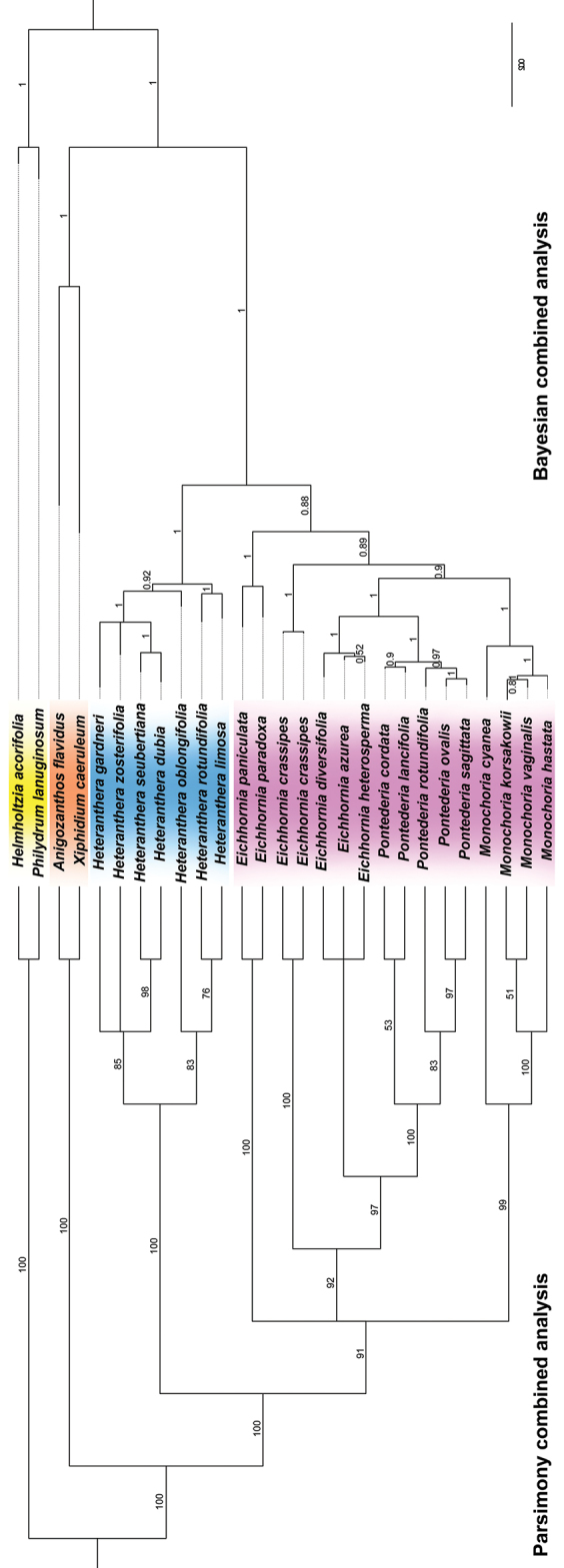
Majority-rule tree recovered for the parsimony and Bayesian analysis of the combined morphological + plastid dataset. Yellow: Philydraceae. Orange: Haemodoraceae. Blue: *Heterantheras.l.* Pink: *Pontederias.l.*

Topologies produced by MP and BI analyses, based on the combined plastid + morphology datasets, were highly congruent and provided higher support for more clades than the results based on independent datasets (Fig. 3). Thus, based on the combined plastid + morphological datasets (1858 analysed characters, of which 353 were variable and 140 parsimony-informative), the maximum parsimony analysis found 24 trees (CI=0.6471, RI=0.7858) whose MRC presented 23 highly supported clades (BSP 75%). The combined Bayesian analysis recovered a fully resolved tree with 25 mostly well-supported clades (>PP 95%) (Fig. 3). The topology recovered for the Bayesian combined analysis (Fig. 3) is almost identical to the one recovered for the plastic dataset (Fig. 2), differing in only very small details. On the other hand, the Parsimony combined analysis recovers *E.crassipes*, *Pontederias.s.* and *Eichhornias.s.* in a well-supported clade, with this clade being recovered in a polytomy together with the *E.paniculata* group and *Monochoria*.

## Discussion

### Phylogenetics of Pontederiaceae

The topologies recovered from the combined plastid and the total evidence datasets strongly corroborate the bi-generic circumscription of Pontederiaceae suggested by Pellegrini (2017a). They are also congruent with previous phylogenetic studies using molecular and/or combined datasets (Graham and Barrett 1995; Barrett and Graham 1997; Graham et al. 1998, 2002; Ness et al. 2011) and partially congruent with the morphologically based phylogenetic tree of Eckenwalder and Barrett (1986). The phylogenetic tree recovered by Kohn et al. (1996) differs greatly from our results and all previous studies due to part of the polyphyletic *Eichhornia* being recovered as sister to *Heterantheras.l.* Most molecular studies in the family (Graham and Barrett 1995; Barrett and Graham 1997; Graham et al. 1998, 2002; Ness et al. 2011) recover a well-supported Pontederiaceae, divided into two main lineages, corresponding to a well-supported *Heterantheras.l.* (*sensu*Pellegrini 2017a) and poorly-supported *Pontederias.l.*; using *ndhF*, *rbcL*, plus a restriction-site in the chloroplast genome in Graham et al. (1998, 2002) and five nuclear gene families recovered employing an expressed sequence tag (EST) study by Ness et al. (2011). As in previous studies (Graham and Barrett 1995; Barrett and Graham 1997; Graham et al. 1998, 2002; Ness et al. 2011), we recover *Pontederias.l.* arranged in five main lineages, each representing a well-supported morphological group (i.e. *Eichhorniapaniculata* group, *Monochoria*, *E.crassipes* group, *Eichhornias.s.* and *Pontederias.s.*). The monophyly of *Heterantherasensu*Pellegrini (2017a) is indisputable and the inclusion of *Hydrothrix* and *Scholleropsis* in *Heteranthera* was strongly corroborated.

### Morphology and systematics of Pontederiaceae

The monophyly of Pontederiaceae was rarely, if ever, questioned by previous authors. Perhaps for this reason, little attention was ever given to the family’s putative morphological synapomorphies. Amongst the 18 morphological synapomorphies recovered for Pontederiaceae, one was previously suggested by Arber (1925; i.e. with xylem and phloem alternate near the centre of the blades, plus xylem abaxial and phloem adaxial near the margins), three were suggested by Simpson (1987, 1990; i.e. late bifacial and ligulate leaves and bisulcate pollen grains) and four were suggested by Simpson and Burton (2006; absence of fibrillar tannin cells in the perianth and presence of aerenchymatous tissue in the receptacle, perianth and ovary walls). Nonetheless, the peculiar involute ptyxis where the blade of the new leaf encloses the petiole of the preceding leaf, non-equitant leaves, sessile leaves early-deciduous, inflorescence deflexed at post-anthesis and in fruit, sessile flowers, perianth connate producing a conspicuous tube and the presence of an anthocarp, are suggested here for the first time as synapomorphies for Pontederiaceae.

Almost, if not all, leaf synapomorphies recovered for Pontederiaceae seem to be directly correlated. These characters seem to be related to the adaptive shift to a completely aquatic lifestyle in the family and an adaptation to changes in water level. The leaves of Pontederiaceae are characteristically dimorphic, being morphologically divided into sessile and petiolate leaves (Horn 1988). Leaf dimorphism is widely distributed across the Embryopsida, being generally related to changes in function (e.g. reproductive leaves in ferns), growth form (e.g. juvenile and mature leaves of *Monstera* spp.) or environmental changes (Allsopp 1965). The dimorphic leaves of Pontederiaceae seem to fit the latter situation, since the petiolate leaves are always floating or aerial, while the ribbon-like or acicular sessile leaves are the first type produced by the germinating plantlet and seen to be an adaptation to the aquatic environment. Furthermore, the presence of a petiole greatly helps to keep the leaves at or above the water level, through cell elongation in the petiolar region. This strategy can be easily observed in several distantly related aquatic plant families (e.g. Alismataceae, Asteraceae, Cabombaceae, Haloragaceae, Nymphaeaceae, Onagraceae, Ranunculaceae etc.; Allsopp 1965; Sculthorpe 1967; Cook 1996). The peculiar vascular bundle arrangement observed in Pontederiaceae is exclusive to the family and few other monocots (Arber 1925). This feature seems to be a result of the reversion from abaxialised unifacial leaves to bifacial leaves, which, according to Simpson (1990), might be related to the adaptive shift and radiation to an aquatic lifestyle in the family. The remaining closely related families (i.e. Haemodoraceae and Philydraceae) possess consistently abaxialised unifacial leaves, with blades ranging from cylindrical, terete, laterally compressed to more rarely plicate (Simpson 1990, 1998; Hamann 1998). Nonetheless, the evolutionary relevance of bifacial leaves is significantly harder to infer, since unifacial leaves are noticeably common in several aquatic plants. The reversal from equitant to alternate leaves seems to be a by-product from the reversion from unifacial to bifacial leaves. As aforementioned, the involute ptyxis in Pontederiaceae is extremely unusual, since the blade of the new leaf encloses the petiole of the preceding leaf. This feature is also unique in the Angiosperms and is easily observed in most species in the family but is especially obvious in *E.crassipes* (Fig. 7C). This feature might also be related to the adaptive shift and radiation to a completely aquatic lifestyle in Pontederiaceae, being most likely a result of the reversion to bifacial leaves. Developmental studies focusing on the ontogeny of the leaves in Pontederiaceae, in comparison to some members of Haemodoraceae and Philydraceae, might help us better understand the mechanics of the reversal from unifacial to bifacial leaves in the family and how this shift might have affected general leaf morphology and the appearance of novel structures such as the ligule.

As aforementioned, the leaves of Pontederiaceae are dimorphic, with both sessile and petiolate leaves being produced at different times in the plants’ life. Sessileleaves represent the plesiomorphic state and are the first ones produced after seed germination. They vary in number from 5–many per plant and allow plants to become established in a submersed habitat (Horn 1988). The sessile leaves can range from early-deciduous to persistent in mature plants, while in some species of *Heterantheras.l.*, petiolate leaves are never or very rarely produced (Horn 1985, 1988; Eckenwalder and Barrett 1986). The petiolate leaves are produced at posteriori and are considered the mature leaf type in the family. The initial petiolate leaves are morphologically plastic, allowing for a transition from a submersed to an immersed environment. This plasticity, coupled with the elongation of the stem, allows Pontederiaceae plants to successfully develop to and at the water surface (Horn 1988). In *Heterantheras.l.*, the sessile leaves suffer a reversion from distichously to spirally arranged, producing the characteristic basal rosettes in the juvenile phase of many *Heteranthera* species (Horn 1988). Thus, early-deciduous sessile leaves and early production of petiolate leaves give a clear adaptive advantage to the Pontederiaceae, enabling them to tolerate a wide variation in water depth during their development, also allowing juvenile plants to successfully reach mature emergent or floating growth-forms (Horn 1988). This might have ultimately allowed the diversification of Pontederiaceae and their complete invasion of the aquatic environment.

**Figure 4.**
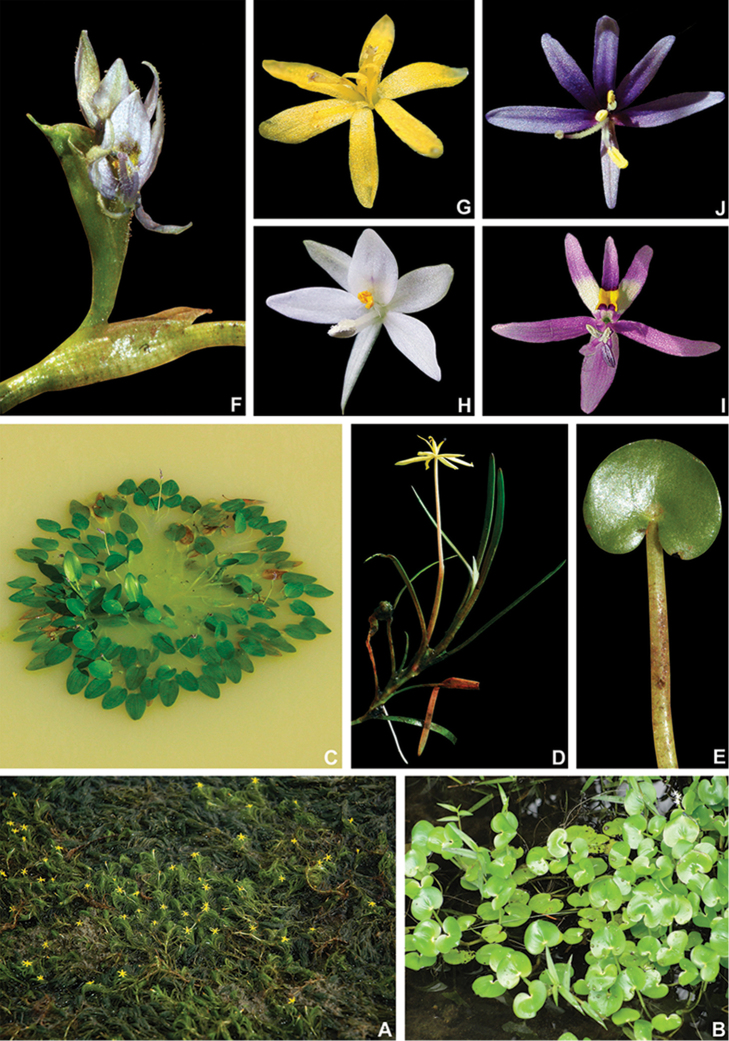
*Heteranthera* Ruiz & Pav. **A–D** habit: **A** emerged and flowering population of *H.gardneri* (Hook.f.) M.Pell. during the dry season **B** floating specimen of *H.reniformis* Ruiz & Pav. **C** emergent habit with floating and emerged leaves of *H.rotundifolia* (Kunth) Griseb. **D** habit of *H.dubia* (Jacq.) MacMill., showing the persistent sessile leaves E petiolate leaf of *H.pumila* M.Pell. & C.N.Horn, showing the lack of a pulvinus **F** Ligule and inflorescence of *H.pumila***G–J** flowers: **G** pseudanthium of *H.gardneri***H***H.reniformis***I***H.rotundifolia***J***H.zosterifolia* Mart. **A** by A.P. Fontana **B, H** by C.N. Horn **C, I** by A. Popovkin **D** by S.R. Turner **E, F** by M.O.O. Pellegrini **G** by C.P. Bove and **J** by S.S. Oliveira.

The presence of a leaf sheath projection is striking in Pontederiaceae, with its morphology being relevant to the systematics of the family. Ligules and ligule-like structures are recorded for several members of Embryopsida, being especially common in some lycophytes (i.e. Selaginellales and Isoëtales) and several monocots (i.e. Alismatales, Arecales, Asparagales, Commelinales, Dioscoriales, Poales and Zingiberales) (Kubitzki 1998; Rudall and Buzgo 2002; Kellogg 2015). Despite possessing the same name, there is no evidence supporting the homology of these structures between lycophytes and monocots and not even between different groups within the monocots (Rudall and Buzgo 2002). The definition and characterisation of ligules in monocots has varied greatly depending on the author, having Poaceae as their main focus. These authors have proposed three distinct definitions for ligules: (1) a subtype of stipule (Bischoff 1834; Regel 1843; Lubbock 1891, 1895; Arber 1925); (2) a structure of mixed origin between stipules and petioles (Glück 1901; Majumbdar 1956); and (3) an avascular projection of the leaf-sheath, situated between the leaf-sheath and the blade (Colomb 1887; Philipson 1935; Dahlgren et al. 1985; Chaffey 1994; Rudall and Buzgo 2002). In Commelinid monocots, ligules and ligule-like structures are recorded for Arecales (i.e. the hastulae present is some Arecaceae leaves), several families of Poales (e.g. Cyperaceae, Joinvilleaceae, Juncaceae, Poaceae, Restionaceae), Commelinales (exclusively in Pontederiaceae) and Zingiberaceae (i.e. Costaceae and Zingiberaceae) (Kubitzki 1998; Rudall and Buzgo 2002; Kellogg 2015). As aforementioned, ligules and ligule-like structures in Commelinales seem to be restricted to Pontederiaceae and are unknown to any of the other four families of the order (Kubitzki 1998; Rudall and Buzgo 2002; Pellegrini pers. obs.). These structures might also be a result of the reversion from unifacial leaves to bifacial leaves or even an independent adaptation to the aquatic lifeform in the family. In the unifacial-leaved clade, composed by Philydraceae (Haemodoraceae+Pontederiaceae), Pontederiaceae is the only exclusively aquatic family and also the only one to possess ligule-like structures (Figs 4F, 6C, 7C, 9E), dimorphic leaves, petiolate leaves and bifacial leaves. Nonetheless, ontogenetic studies are necessary to understand the origin of these structures in the family. In Pontederiaceae, these ligule-like structures have been treated under different names according to the authors, having been named stipules (Schwartz 1926), ligules (Castellanos 1958; Pellegrini and Horn 2017), ochreas (Rutishauser 1999) or simply as leaf-sheath projections (Pellegrini 2017a). Different names have also been applied by the same author, depending on the development and shape of these structures (i.e. Cook 1998). Regardless of the name adopted for these ligule-like structures in Pontederiaceae, their systematic and taxonomic relevance is undeniable. As aforementioned, this structure is recovered as synapomorphic for the family. Alternatively, within Pontederiaceae, the morphology of this structure can be easily used to define the two clades recovered in phylogenetic studies. *Pontederias.l.* can be easily characterised by it mainly truncate ligules, being rarely flabellate (i.e. *E.crassipes*); while *Heterantheras.l.* can be characterised by its 2–several-parted ligules.

Out of the reproductive synapomorphies recovered by us for Pontederiaceae, some of them seem to be related to pollination, while the others seem to be related to fruit dispersal. Sessile flowers are recovered by us as a synapomorphy of Pontederiaceae, with the sole reversion occurring in *Monochoria*. This character seems to be directly related to another reproductive synapomorphy for the family (i.e. perianth connate to part of the receptacle and the filaments producing a conspicuous tube). Pedicel and floral tube length seem to be inversely correlated, with tube elongation helping with the floral display by elevating the perianth lobes. Added to that, the contraction of the pedicel might also provide extra stability for heavier floral visitors that require landing platforms in order to properly visit flowers (e.g. butterflies). Alternatively, the reversion from sessile to pedicellate flowers in *Monochoria* might have played a key role, by giving flowers the needed mobility in order to avoid floral damage during buzz pollination (Wang et al. 1995). Bisulcate pollen grains are rather rare in the monocots, being recorded for only a handful of families, such as: Araceae (Grayum 1992), Arecaceae (Harley and Baker 2001), Dioscoreaceae (Caddick et al. 1998), Iridaceae (Rudall and Wheeler 1988) and Velloziaceae (Halbritter and Hesse 1993). Of the aforementioned families, only Arecaceae (Arecales) is a member of the Commelinid monocots and it is but distantly related to Pontederiaceae (Saarela et al. 2008; Hertweck et al. 2015; APG IV 2016). In Haemodoraceae, Simpson (1983) recorded the occurrence of biporate pollen grains in some genera from subfamily Conostylidoideae. Nonetheless, Simpson (1987, 1990) considers the biporate pollen grains in Haemodoraceae not homologous to the bisulcate pollen grains in Pontederiaceae. This view is also shared by us in the present study.

**Figure 5.**
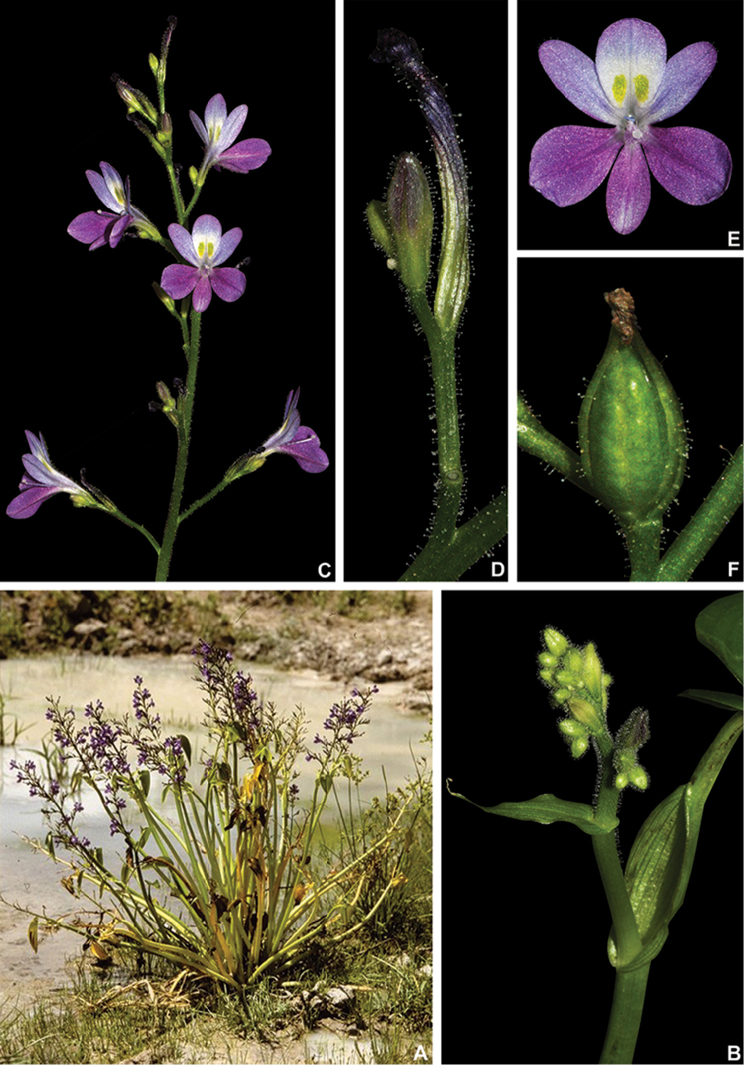
Pontederiasubg.Cabanisia (Klotzsch *ex* Schltdl.) M.Pell. & C.N.Horn. **A** habit **B–C** inflorescence: B young inflorescence, showing the inflated leaf-sheath and flat basal bract with caudate apex **C** mature inflorescence showing the pedunculate cincinni with elongate internodes **D** detail of a cincinni, showing (from left to right) an immature floral bud, a pre-anthesis floral bud and a post-anthesis flower **E** front view of a flower F detail of an immature capsule, showing the ridged anthocarp. All photos of *P.paniculata* Spreng.; **A** by C. Willig & L. Nusbaumer, remaining photos by M.O.O. Pellegrini.

**Figure 6.**
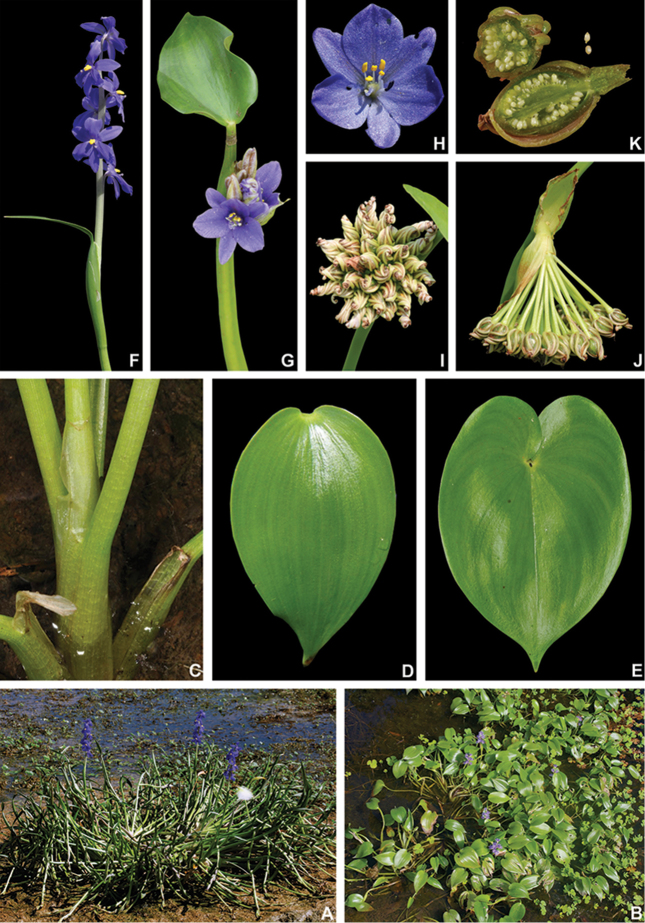
Pontederiasubg.Monochoria (C.Presl) M.Pell. & C.N.Horn. **A–B** habit: **A** paludal habit of *P.australasica* (Ridl.) M.Pell. & C.N.Horn **B** paludal habit of *P.cyanea* (F.Muell.) M.Pell. & C.N.Horn **C** ligule of *P.vaginalis* Burm.f., showing the truncate apex **D–E** petiolate leaf-blades: **D** blade of *P.cyanea*, showing the lack of a posterior division **E** blade of *P.vaginalis*, showing the presence of a posterior division **F–G** inflorescences: **F** inflorescence of *P.australasica*, showing the developed main axis **G** inflorescence of *P.plantaginea* Roxb., showing the contracted main axis **H** front view of a flower of *P.korsakowii* (Regel & Maack) M.Pell. & C.N.Horn **I–J** inflorescences at post-anthesis: **I** erect inflorescence of *P.hastata* L. bearing flowers at post-anthesis J infructescence of *P.hastata*, showing the deflexed posture and the elongated pedicels K sections of immature capsules of *P.vaginalis*, showing developing seeds. **A, F** by M. Barritt **B** by R. Cumming **C, E, K** by P.B. Pelser & J.F. Barcelona **D** by A. & S. Pearson **G** by D. Valke **H** by Ashitaka-f Studio and I & J by Cerlin Ng.

The first synapomorphy related to diaspore dispersal is the deflexed position of the inflorescence at post-anthesis and in fruit. This shift in the inflorescence position during fruit development will almost certainly allow the mature fruits to reach the water after their maturity. The deflexed inflorescences also elongate in length, which ultimately places the maturing fruits at or under the water surface. This seems to be the first step in diaspore dispersion in most species of Pontederiaceae. The following adaptations are related to increasing the floatation period of the diaspores. The first and most obvious seems to be the presence of an anthocarp. According to Spjut (1994), an anthocarp is a type of fruit which possesses attached and developed floral parts that aid in its dispersal. It is more commonly recorded for plants with inferior ovaries, but it is not exclusive to them (Spjut 1994). In Commelinales, all fruits have persistent perianth parts, but only in Pontederiaceae does an enlarged perianth actively aid in the dispersal of the diaspores (Pellegrini, pers. observ.), with *Tradescantiazanonia* (L.) Sw. (Commelinaceae) being an exception (Pellegrini 2017b; Pellegrini and Faden 2017). In Pontederiaceae, the anthocarp seems to be related to hydrochoric dispersion, which is also supported by the remaining synapomorphies for the family (i.e. presence of aerenchymatous tissue in the receptacle, perianth and ovary walls). The anthocarp is especially developed with thick aerenchymatous tissue in *Monochoria*, *Pontederias.s.* and in the *E.paniculata* group (Lowden 1973; Cook 1989, 1998; Simpson and Burton 2006; Pellegrini, pers. observ.; Figs 5F, 6K & 9K), that provides long flotation periods for the diaspores (i.e. around 15 days; Barrett 1988). In the remaining lineages of Pontederiaceae (i.e. *Heterantheras.l.*, *E.crassipes* group and *Eichhornias.s.*), the anthocarp is thin, probably resulting in a much shorter flotation period (i.e. probably around 24h), with seeds being secondarily dispersed by other biotic and/or abiotic means (Barrett 1978; Pellegrini and Horn, pers. observ.). In the closely-related Haemodoraceae and Philydraceae, the perianth is also connate, producing a characteristic hypanthium and partially to completely persistent in fruit (Hamann 1998; Simpson 1998). Nonetheless, they do not aid in the dispersal of diaspores, since in all species, the persistent perianth is only marcescent and does not develop during fruit development, being ultimately torn open by the mature fruit (Pellegrini, pers. observ.). These observations are also supported by the complete lack of aerenchymatous tissues in floral organs of both families, with aerenchyma being recorded only in the septae of the hydrochoric Philydraceae (Simpson and Burton 2006). In Commelinaceae and Hanguanaceae, the persistent perianth also does not develop during fruit maturation; with the exception of *Buforrestia* C.B.Clarke (Commelinaceae), where the persistent sepals are as long as, or longer than, the mature capsule (Bayer et al. 1998; Faden 1998). Nonetheless, the perianth of *Buforrestia* does not seem to aid in the dispersion of the diaspores, since the perianth only loosely involves the capsules, which remain attached to the pedicel and dehisce at maturity (Pellegrini, pers. observ.). In Hanguanaceae, the fruits consist of variously coloured berries that detach from the persistent sepaloid perianth and are most probably zoochoric (Bayer et al. 1998). On the other hand, in Commelinaceae, the fruits are primarily dehiscent capsules (rarely indehiscent capsules or berries), that do not rely on the persistent sepals for dispersion, with fruits or seeds being autochoric or more rarely zoochoric (Pellegrini and Faden 2017).

### Systematics and characterisation of *Pontederia* s.l.

All 18 synapomorphies recovered by us for *Pontederias.l.* are suggested here for the first time. Sand-binding roots were recovered by Smith et al. (2011) as plesiomorphic for Haemodoraceae and probably for all Commelinales, despite the authors’ not sampling Hanguanaceae in their analysis. These sand-binding roots produce specialised hairs that bind soil, especially larger sand crystals, creating a protective layer that envelops the roots (Smith et al. 2011). These authors also state that all studied specimens of Philydraceae and Pontederiaceae had non-sand-binding roots, in contrast to Haemodoraceae. On the other hand, sand-binding roots are commonly observed in several lineages of Commelinaceae, but especially in species growing in dry environments (Smith et al. 2011; Pellegrini, pers. observ.). After several field studies and cultivation of several species of Pontederiaceae, we have observed that all species of *Heterantheras.l.* possess water-binding (i.e. mucilaginous) roots, while the absence of an external mucilage layer on the roots was characteristic of *Pontederias.l.* The water-binding roots of *Heterantheras.l.* are most probably not homologous to the sand-binding roots in the order, since they do not seem to have specialised hairs, like those described for Haemodoraceae (Smith et al. 2011). The mucilage layer seems to be produced by the secretion of chemical compounds near the root apex which polymerises in contact with water (Pellegrini, pers. observ.). Nonetheless, further anatomical and histochemical studies are needed to better understand this feature.

The presence of leaves with pulvinate petioles in *Pontederias.l.* is easily observed in the field, since most pulvini are lighter or darker than the rest of the petiole. On the other hand, in dried specimens, this difference in colouration is only sometimes maintained, making this character not always obvious to untrained eyes. Added to that, the pulvini in *Pontederias.l.* are seldom swollen, as would be expected in most eudicot plants with articulated leaves. Nonetheless, this feature seems to be key for the emergent and floating species, especially the perennial ones, since they are subjected to the greatest amount of environmental variation. Floating species like *E.crassipes* are easily dragged by water currents, forcing all leaf-blades to change their position in order to better absorb sunlight. Perianth-coiling at post-anthesis seems to be poorly documented in the literature for most Angiosperm families and more so in the monocots. It is known to occur in the monocots only in the distantly related Bromeliaceae (Poales), being characteristic to some genera of subfamilies Pitcairnioideae and Puyoideae (Smith et al. 1998; Hornung-Leoni and Sosa 2008). In Commelinales, the persistent perianth is marcescent in Philydraceae, Haemodoraceae and Hanguanaceae, while in Commelinaceae, the sepals are marcescent and the petals are deliquescent (Pellegrini, pers. observ.). In Pontederiaceae, the perianth in *Heterantheras.l.* is also marcescent at post-anthesis, only loosely enclosing the developing capsule. In *Pontederias.l.*, the perianth is either spirally-coiled or revolute at post-anthesis, tightly enclosing the developing fruit, with two independent shifts to deliquescent perianths loosely enclosing the developing fruit (i.e. *E.crassipes* and *Eichhornias.s.*). This might be related with increasing long-distance diaspore dispersal in the rooted species, with the anthocarp ridges possessing aerenchymatous tissue in most species. This character seems to greatly increase the dispersion range of most *Pontederias.l.* lineages that, unlike *E.crassipes* and *Eichhornias.s.*, are not easily vegetatively dispersed by the fragmentation of floating stems. In *E.crassipes*, the plants are free-floating and can easily disperse in waterbodies with moving waters, while in *Eichhornias.s.*, the plants have elongated stems, which possibly help diaspores to disperse further away from the mother plant’s base, thus decreasing parental/offspring competition.

Tristyly is an extremely rare type of heterostyly, recorded for a handful of families, only two being monocots (i.e. Amaryllidaceae and Pontederiaceae; Barrett 1993). According to Kohn et al. (1996), tristyly evolved only once in Pontederiaceae. As aforementioned, in Kohn et al. (1996), they recover part of the polyphyletic *Eichhornia* as sister to *Heterantheras.l.* and tristyly as a synapomorphy for Pontederiaceae as a whole, with four reversions to homostyly. However, we recover tristyly as a synapomorphy of *Pontederias.l.* alone, with only two reversions to homostyly. In *E.diversifolia* (Vahl) Urb. and *E.natans* (P.Beauv.) Solms, the flowers seem to be consistently pseudo-homostylous, which could be related to miniaturisation connected with these species’ floating growth-form (Barrett 1988). In *Monochoria*, there is a shift from tristyly to enantiostyly (i.e. two different types of heterostyly; Barrett 1993), that could be easily explained by the shift in the group’s pollination syndrome. *Monochoria* species are enantiostylous, lack septal nectaries and exclusively offer pollen as a floral reward (Wang et al. 1995) and this, most likely, is connected with the buzz pollination syndrome of their flowers. Furthermore, poricidal, basifixed, polymorphic anthers are typical to buzz-pollinated flowers (Cook 1989; Wang et al. 1995). This shift from nectar-flowers to pollen-flowers seems to be the main cause of the peculiar floral morphology and loss of tristyly in *Monochoria*.

In Pontederiaceae, three different patterns in perianth-lobe shape can be observed: (1) perianth lobes all equal, thus producing an actinomorphic perianth (e.g. *H.dubia*); (2) equal to subequal in the same whorl, producing either actinomorphic or zygomorphic perianths, depending on the presence of a nectar guide [e.g. actinomorphic in *M.hastata* (L.) Solms and zygomorphic in *E.crassipes*]; and (3) unequal perianth lobes, with more than one morph in the same whorl, producing strongly zygomorphic perianths (e.g. *H.gardneri*). In Commelinales, the perianth lobes pattern seems to be extremely variable, being equal in the same whorl in Hanguanaceae, unequal in Philydraceae (due to the fusion of three posterior lobes) and variable in Commelinaceae and Haemodoraceae (Pellegrini, pers. observ.). In Commelinaceae, sepals are almost invariably different from the petals, except in *Palisota* Rchb. *ex* Endl. in which the sepals are characteristically petaloid (Faden 1998). Furthermore, both sepals and petals can range from equal to unequal, producing strongly zygomorphic flowers (e.g. *Aneilema* R.Br., *Commelina* L., *Polyspatha* Benth.; Faden 1998). In Haemodoraceae, there is much variation in the shape of the perianth lobes (Simpson 1990, 1998). Nonetheless, equal perianth lobes seem to be plesiomorphic in the monocots (Sauquet et al. 2017; Stevens 2001–onwards) and dominant in the family, being recorded for 11 out of 14 genera (Pellegrini, pers. observ.). Thus, equal to subequal lobes in one perianth whorl (the apices are obtuse to round) is recovered by us as a homoplastic synapomorphy for *Pontederias.l.* (Fig. 1). The perianth in *Pontederias.l.* ranges from campanulate to infundibuliform to hypocrateriform, while in *Heterantheras.l.*, it is almost exclusively tubular, a distinctive synapomorphy for the latter genus. The only exception is *H.gardneri*, which possesses an infundibuliform perianth and which might be explained by miniaturisation. In Philydraceae, the perianth is consistently infundibuliform, while the perianth in Haemodoraceae shows great plasticity, depending on the genus, ranging from flat to hypocrateriform to tubular to the peculiar split and falcate perianth of *Anigozanthos* (Simpson 1990, 1998).

### Systematics and characterisation of the five main lineages of *Pontederia* s.l.

Out of the four synapomorphies recovered for the *E.paniculata* group, two had been previously proposed by Eckenwalder and Barrett (1986; annual life cycle) and Barrett and Graham (1997; annual life cycle and the absence of clonal reproduction). All currently accepted species in this group are known to inhabit seasonal and, generally, short-lived waterbodies. Thus, the annual life cycle and the absence of clonal reproduction are more than expected. However, all previous studies in the family failed to notice the peculiarly inflated sheath of the leaf subtending the inflorescence and the flat basal bract (Fig. 5B). These characters are easily observed in *E.paniculata* and *E.meyeri*, due to their elongated inflorescences, while in *E.paradoxa*, the inflorescence has its internodes greatly contracted, thus making the flat basal bract extremely hard to observe, especially in dried specimens.

*Monochoria* comprises species with extremely autapomorphic morphology, being traditionally grouped based on their: pedicellate, actinomorphic and enantiostylous flowers, basally connate perianth and its basifixed and poricidal anthers (Cook 1989, 1998). Due to its enantiostylous flowers and basifixed anthers, *Monochoria* has traditionally been considered closely related to *Heteranthera* (Eckenwalder and Barrett 1986; Cook 1998). Nonetheless, molecular data provide strong support that *Monochoria* is instead sister to the clade composed of *E.crassipes*, *Eichhornias.s.* and *Pontederias.s.* (Graham and Barrett 1995; Kohn et al. 1996; Barrett and Graham 1997; Graham et al. 1998, 2002; Ness et al. 2011; this study). Aside from the six aforementioned synapomorphies, *Monochoria* is also supported in our present analysis by other six characters. Out of these characters, only the basal bract with a caudate apex was previously described as characteristic of *Monochoria* by Cook (1989). The presence of an inflated sheath in the leaf subtending the inflorescence, flat basal bract and fistulose main axis are shared between the *E.paniculata* group and *Monochoria* and are most likely plesiomorphic for *Pontederias.l.* The caudate apex in the basal bract is observed in all species of *Monochoria*. Nonetheless, *M.korsakowii* can also present a leaf-like basal bract (Cook 1989). The actinomorphic perianth is a result of the loss of the nectar guide in this lineage which, as aforementioned, is directly related to the shift in pollination syndrome in the group. Additionally, other four floral modifications in *Monochoria* seem to be associated with this shift in the group’s pollination syndrome: (1) pedicellate, actinomorphic and enantiostylous flowers; (2) basally connate perianth (which helps to expose the stamens and allows the bees to properly visit the flowers); (3) unequal, basifixed and poricidal anthers; and (4) the loss of septal nectaries. The presence of a petalo-staminal tube is also unique in the family and most probably is the result of the reduction of the length of the hypanthium. Finally, the thickened and ridged anthocarps are also observed in the *E.paniculata* group and *Pontederias.s.*, being directly related to the fruits primary hydrochoric dispersal syndrome (see comment above).

Despite being well-known, *E.crassipes* possesses the most peculiar vegetative morphology in the polyphyletic *Eichhornia* and one of the most peculiar in the family as a whole. It is so peculiar that specimens are easily identified, even when lacking any reproductive structures (Pellegrini and Horn, pers. observ.). It is the only species in the family to possess a free-floating growth form, the only one to produce stolons and the only one to possess inflated petioles. Nonetheless, one of the most peculiar characters in *E.crassipes* has been greatly disregarded by most specialists in the family. Castellanos (1958) was one of the first to properly describe and illustrate the flabellate ligules of *E.crassipes*. All synapomorphies recovered for *E.crassipes* seem to be directly related to its peculiar free-floating growth form, which also enabled it to become the most troublesome weed of the world (Gopal and Sharma 1981). The morphology of *Eichhornias.s.* is clearly a result of its floating growth form and the tendency of these plants to grow in deeper water bodies. The late-deciduous sessile leaves (sometimes persistent for most of the plant’s adult life) are characteristic of this group, but especially striking in *E.diversifolia*, hence its name. This protraction of the submerged phase seems to give the species in this clade a clear developmental advantage by helping them to reach the water surface and produce enough petiolate leaves to allow them to float properly. Furthermore, the even arrangement of the petiolate leaves along the mature stem might help provide the needed stability to the elongated floating stem.

From all the recovered clades in *Pontederias.l.*, *Pontederias.s.* goes hand-in-hand with *Monochoria* in the number of reproductive synapomorphies. Out of the eight recovered synapomorphies for this clade, six are reproductive, with only the presence of epithelial cells in the septae, which are shared with *Monochoria*, being homoplastic. All the remaining five reproductive synapomorphies are directly correlated, but their evolutionary chronology is much harder to infer. The most parsimonious view is probably that all characters were triggered concomitantly by the appearance of the pseudomonomerous ovary, which caused the change in placentation morphology and ovule number. The abortion of most of the gynoecium might have caused a key shift in the reproductive strategy in this lineage from investing in a great number of small seeds with little chance of reaching maturity, to investing into a single big seed with a good amount of provision and guaranteeing that it has a greater chance of reaching maturity. The smooth testa seems to be a simple byproduct of negative selection of ornamentation, since the seeds stopped being individually dispersed with the change of reproductive strategy. Finally, the achene gives this lineage a great evolutionary advantage since it is easily dispersed by water, with a long floatation period due to its thick parenchymatous walls. Furthermore, many species also possess complex ornate achenes, with teeth and spikes that efficiently stick to fur, feathers, fabric etc., most likely having animals as their primary dispersers (Pellegrini, pers. observ.).

### Taxonomy

With the present recircumscription of *Pontederia*, Pontederiaceae now is organised in two monophyletic genera (i.e. *Heteranthera* and *Pontederia*). As stated by Pellegrini (2017a) and corroborated by nine phylogenetic studies (Eckenwalder and Barrett 1986; Graham and Barrett 1995; Kohn et al. 1996; Barrett and Graham 1997; Graham et al. 1998, 2002; Ness et al. 2011; this study), the recognition of two genera seems to be the best and most taxonomically conservative option available, since it avoids the description of new genera and the reestablishment of names that were rarely, if ever, used in any relevant taxonomic or floristic study. Finally, this option makes the differentiation of the two accepted genera easy, using either fresh, liquid or herbarium samples. Thus, the genera of Pontederiaceae can be differentiated using the key below:

### Key to the genera of Pontederiaceae

**Table:** 

1	Sessileleaves spirally-alternate, petiolate leaves sometimes present in mature specimens, when present non-pulvinate, blade membranous; inflorescence reduced to a solitary cincinnus; stamens (1–)3, staminodes sometimes present, septal nectaries absent, stigma unevenly trilobate	**Heteranthera Ruiz & Pavón** (Fig. 4)
–	Sessileleaves distichously-alternate, petiolate leaves always present in mature specimens, pulvinate, blade chartaceous to coriaceous; inflorescence a 2–many branched thyrsi (rarely reduced to a solitary flower); stamens 6, staminodes absent, septal nectaries present (if absent, then flowers pedicellate and anthers poricidal), stigma capitate or trilobate, rarely trifid	***Pontederia* L.** (Figs 5–9)

#### Pontederia

Taxon classificationPlantaeCommelinalesPontederiaceae

L., Sp. Pl. 1: 288. 1753.

**Figure 7.**
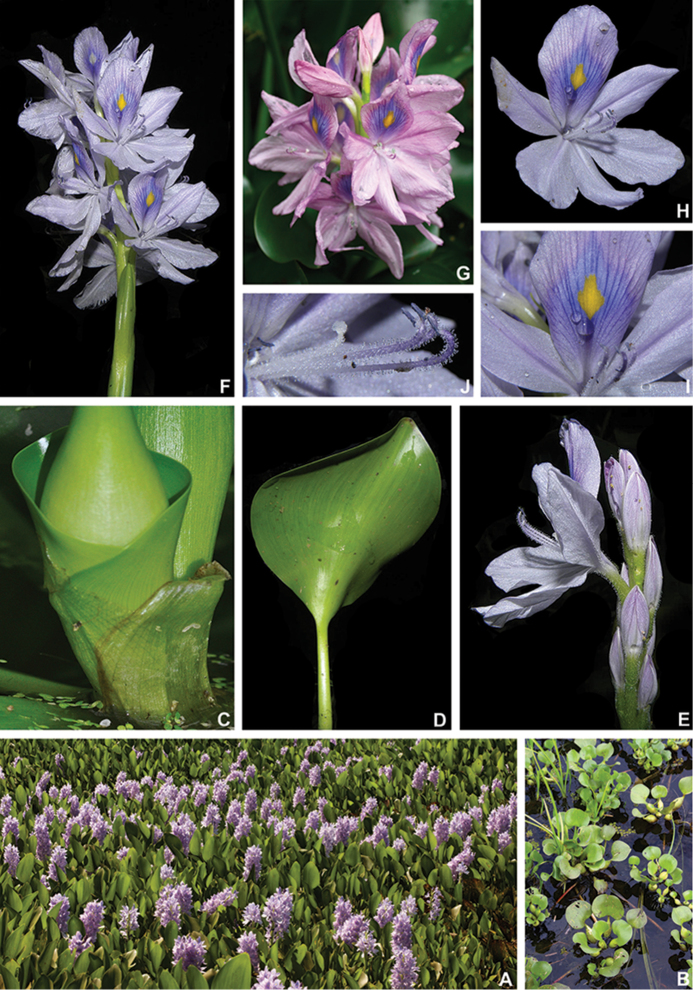
Pontederiasubg.Oshunae M.Pell. & C.N.Horn. **A–B** habit: **A** dense population of the pink-flowered form **B** detail of a population, showing the free-floating rosettes, stolons and inflated petioles **C–D** petiolate leaves: **C** blade **D** detail of a young leaf showing its blade enclosing the inflated petiole of the presiding leaf **E–G** inflorescence: **E** young inflorescence of a lilac-flowered form **F** inflorescence of a lilac-flowered form at anthesis G inflorescence of a pink-flowered form at anthesis **H–J** flowers: **H** oblique view of a lilac flower **I** detail of the nectar guide J detail of the androecium and gynoecium showing the glandular hairs. All photos of *P.crassipes* Mart.; **A** by C. Willig & L. Nusbaumer **B** by O. Gaubert **C** by K. Pritchard & S.A. Harris, **D–F, H–I** by R. Aguilar and **G** by M.O.O. Pellegrini.

**Figure 8.**
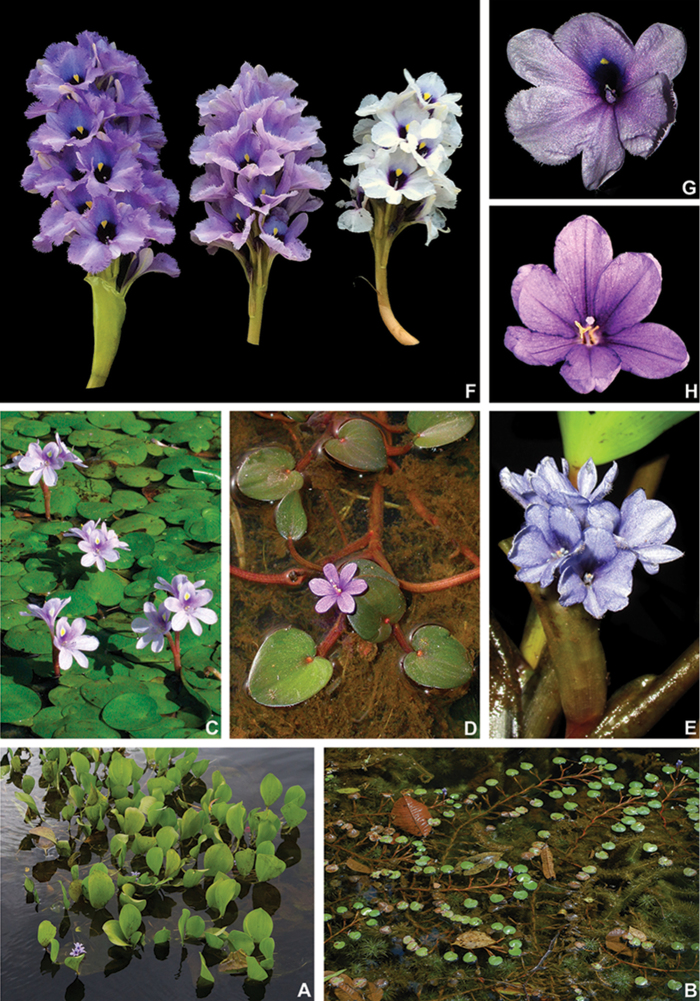
Pontederiasubg.Eichhornia (Kunth) M.Pell. & C.N.Horn. **A–B** habit: **A** habit of *P.heterosperma* (Alexander) M.Pell. & C.N.Horn, showing the emerged petiolate leaves **B** habit of *P.diversifolia* (Vahl) M.Pell. & C.N.Horn, showing the floating petiolate leaves **C–F** inflorescence: **C** 2–3-flowered inflorescences of *P.diversifolia*, showing the flowers with a yellow nectar guide in the posterior perianth lobes **D** 1-flowered inflorescence of *P.natans* P.Beauv., showing the lack of a nectar guide **E** inflorescence of *P.heterosperma*, showing the lack of nectar guides in the posterior perianth lobes **F** morphological variation of inflorescences and perianth colour of *P.azurea* Sw **G** front view of a flower of *P.azurea* H front view of a flower of *P.natans*. **A, B** by O. Gaubert **C** by A.S. Castro **D** by P. Birnbaum **E** by H. Medeiros **F** by L.O.A. Teixeira **G** by M.O.O. Pellegrini and I by T.C. Buruwate.

**Figure 9.**
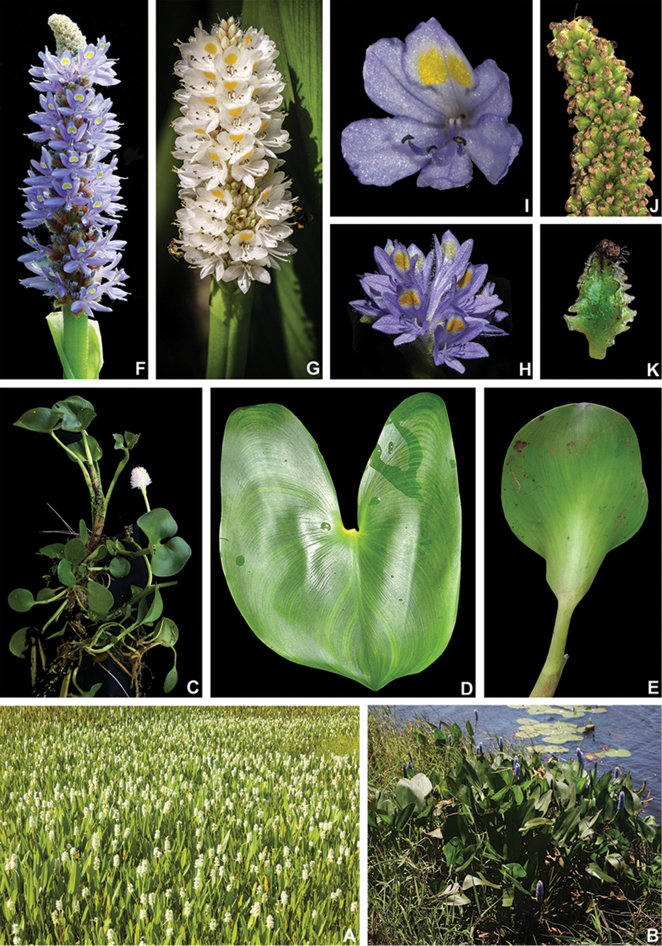
PontederiaL.subg.Pontederia. **A–C** habit: **A** dense population of *P.parviflora* Alexander **B** population of *P.ovalis* Mart. *ex* Schult. & Schult.f. **C** habit of *P.rotundifolia* L.f. **D–E** petiolate leaves: **D** blade of *P.rotundifolia***E** blade of *P.parviflora***F–H** inflorescences: **F** inflorescence of *P.cordata* L., showing flowers with two yellow nectar guides in the posterior perianth lobes **G** inflorescence of *P.parviflora*, showing flowers with a sole yellow nectar guide in the posterior perianth lobes **H** inflorescence of *P.rotudifolia*, showing a lilac-flowered form **I** oblique view of a flower of *P.ovalis***J–K** fruits: **J** detail of the apex of the infructescence of *P.ovalis*, showing the anthocarp with sinuate ridges **K** detail of an achene of *P.cordata*, showing the toothed ridges. **A** by C. Willig & L. Nusbaumer **B, I, J** by M.O.O. Pellegrini **C** by L.O.A. Teixeira, **D, H** by R. Aguilar **E** by M.R. Engels **F** by Ashitaka-f Studio **G** by M.V. Lameiras and **K** by A. Haines.

Figs 5 , 6 , 7 , 8 , 9

##### Type species (designated by Lowden 1973).

*Pontederiacordata* L.

##### Description.

*Herbs* perennial or annual, aquatic to amphibious, erect-emergent, procumbent-emergent or free-floating. *Roots* thin, fibrous or spongy. *Rhizome* short and generally inconspicuous. *Stems* trailing to erect, delicate to spongy, branching at the base, rarely branching at the upper half, rooting at the basal nodes or along the whole stem; internodes reduced to elongate, producing stolons or not. *Sessileleaves* distichously-alternate, congested at the apex of the stem, submerged, deciduous or persistent in mature plants, blades linear to linear-obovate, membranous, rarely chartaceous. *Petiolateleaves* distichously or spirally-alternate, congested at the apex of the stem or evenly distributed along the stem, floating or emergent, ligule truncate or with a flabellate projection; petioles conspicuous, rarely indistinct, inflated or not; blades elliptic to lanceolate or ovate to cordate to reniform or obovate to rounded, chartaceous to coriaceous. *Synflorescence* composed by a solitary main florescence subtended by a vegetative, petiolate leaf. *Main florescences (inflorescences)* axillary or apparently terminal, consisting of a pedunculate, many-branched thyrse, rarely a reduced thyrse; inflorescence leaf with or without an inflated leaf-sheath; basal bract flat or tubular; cincinnus’ bract absent; cincinni (1–3–)4 – many per thyrse, alternate or fascicle-like, 1 – many-flowered, sessile or pedunculate, internodes contracted, rarely elongate; bracteoles absent, rarely present. *Flowers* bisexual, sessile or pedicellate, chasmogamous, pseudo-homostylous or tristylous, enantiostylous, zygomorphic, perianth connate usually forming a tube (hypanthium), rarely only basally fused, campanulate or infundibuliform or hypocrateriform, white to light pink to pink to mauve to pale lilac to lilac to bluish-lilac to purple, lobes 6 (3 outer and 3 inner), elliptic to oblong to obovate, 3 superior and 3 inferior, rarely 5 superior and 1 inferior, the central superior lobe generally with a nectar guide, consisting of 1–2 yellow to green spots, generally surrounded by a dark purple to bluish-purple, rarely white blur, spirally-coiling or revolute at post-anthesis, deliquescent or not; stamens 6, epipetalous, dimorphic (the superior 3 shorter than the inferior 3) or unequal (1 inferior longer with a differently coloured anther), filaments J-shaped or recurved-decurved, terete, glabrous to glandular-pubescent, anthers dorsifixed, sometimes basifixed, rimose or poricidal, oblong to elliptic or sagittate; ovary ellipsoid to oblongoid, glabrous, locules 3, (1–)3 fertile, (1–)multi-ovulate, placentation axial or pendulous, septal nectaries generally present, rarely absent, style J-shaped, glabrous to glandular-pubescent, stigma capitate to trilobate, rarely trifid. *Fruit* a capsule with loculicidal or irregular dehiscence, rarely an achene, ellipsoid to oblongoid to subglobose or ovoid, rarely pyriform, light to medium brown, apiculate due to persistent style base; anthocarp thin or hardened, smooth or ridged, ridges ornamented or not. *Seeds* oblongoid or ellipsoid or subglobose to broadly oblongoid or ovoid or curved narrowly ovoid, brown to light-brown, testa longitudinally conspicuously to inconspicuously winged, rarely smooth, when present, wings membranous and testa also transversally striated between each wing; funiculi generally persistent, hilum punctate; embryotega dorsal, not prominently apiculate, darker than the rest of the seed.

##### Distribution and habitat.

*Pontederia* currently comprises 26 mainly Neotropical species. Almost all Paleotropical species belong to P.subg.Monochoria (C.Presl) M.Pell. & C.N.Horn *comb. et stat. nov.*; except for *P.natans* P.Beauv., which is restricted to Africa and is a member of P.subg.Eichhornia (Kunth) M.Pell. & C.N.Horn *comb. et stat. nov.* Species in *Pontederia* can range from paludal to free-floating plants, thus occurring in a wide range of water bodies, from perennial to temporary, but most commonly in slow or stagnated water.

##### Generic circumscription and infrageneric classification.

The circumscription adopted by us is almost equivalent to the original one proposed by Linnaeus (1753). It differs only by the exclusion of *P.ovata* L., which is currently placed in Marantaceae as a synonym for *Phryniumpubinerve* Blume (Horn and Haynes 1987; eMonocot 2010). Thus, no amendments are necessary for the herein adopted circumscription. We propose the subdivision of *Pontederia* in five monophyletic subgenera, based on the previously published molecular and morphological phylogenies (Eckenwalder and Barrett 1986; Graham and Barrett 1995; Kohn et al. 1996; Barrett and Graham 1997; Graham et al. 1998, 2002; Ness et al. 2011), added to the new morphological and molecular analyses presented by us and data gathered by us while working on the family. Despite being monophyletic, these subgenera are not easily morphologically differentiated, since many of the characters supporting each clade are not always easy to observe, especially in dried specimens. Thus, it is our opinion that a broader sense of *Pontederia* should be accepted, instead of elevating each *Eichhornia* lineage (i.e. the herein proposed subgenera) to the generic rank.

### Key to the subgenera of Pontederia

**Table:** 

1	Basal bract commonly with a caudate apex, rarely leaf-like; flowers pedicellate, enantiostylous, perianth only basally connate, campanulate; stamens with filaments connate forming a petalo-staminal tube, anthers basifixed, poricidal; septal nectaries absent	**Pontederiasubg.Monochoria (C.Presl.) M.Pell. & C.N.Horn** (Fig. 6)
–	Basal bract with an acute to acuminate to aristate apex, rarely caudate; flowers sessile, non-enantiostylous, perianth connate forming a conspicuous tube, infundibuliform or hypocrateriform; stamens with free filaments, anthers dorsifixed, rimose; septal nectaries present	**2**
2	Ovary 1-locular by abortion, fertile locule 1-ovulate, placentation pendulous; fruit an achene, anthocarp hardened, ridges sinuate, toothed or echinate; seeds smooth	**PontederiaL.subg.Pontederia** (Fig. 9)
–	Ovary 3-locular, locules many-ovulate, placentation axial; fruit a capsule, anthocarp thin to thickened, if thickened ridges smooth; seeds longitudinally winged	**3**
3	Herbs procumbent-emergent, stems elongate; sessile leaves late deciduous, rarely persistent in mature plants, petiolate leaves distichously-alternate, evenly distributed along the stem; perianth infundibuliform, style glabrous	**Pontederiasubg.Eichhornia (Kunth) M.Pell. & C.N.Horn** (Fig. 8)
–	Herbs erect emergent or free-floating, stems inconspicuous; sessile leaves early deciduous, petiolate leaves spirally-alternate, congested at the apex of the stem; perianth hypocrateriform, style glandular-pubescent	**4**
4	Herbs stoloniferous; ligule flabellate, petioles generally inflated; inflorescences deflexed post-anthesis and in fruit, emerging from a non-inflated leaf-sheath, basal bract tubular; flowers ca. 4–6 cm diam., perianth loosely enclosing the developing fruit; seeds oblongoid	**Pontederiasubg.Oshunae M.Pell. & C.N.Horn** (Fig. 7)
–	Herbs never producing stolons; ligule truncate, petioles never inflated; inflorescences erect at post-anthesis, emerging from an inflated leaf-sheath, basal bract flat; flowers ca. 2–3 cm diam., perianth tightly enclosing the developing fruit; seeds subglobose to broadly oblongoid	**Pontederiasubg.Cabanisia (Klotzsch ex Schltdl.) M.Pell. & C.N.Horn** (Fig. 5)

#### 1. Pontederia subg. Cabanisia

Taxon classificationPlantaeCommelinalesPontederiaceae

(Klotzsch ex Schltdl.) M.Pell. & C.N.Horn comb. et stat. nov.

urn:lsid:ipni.org:names:77188076-1

Fig. 5

Cabanisia Klotzsch *ex* Schltdl., Abh. Naturf. Ges. Halle 6: 176. 1862. Type species (designated here). Cabanisiacaracasana Klotzsch *ex* Schltdl., nom. illeg. (≡ P.paniculata Spreng.).

##### Description.

*Herbs* perennial or annual, aquatic to amphibious, erect-emergent. *Rhizome* short and generally inconspicuous. *Stems* erect, spongy, branching at the base. *Sessileleaves* early deciduous. *Petiolateleaves* spirally-alternate, congested at the apex of the stem, emergent, ligule truncate, petioles not-inflated, blades cordate to broadly cordate, rarely elliptic to lanceolate or narrowly ovate. *Main florescences (inflorescences)* terminal, sessile or pedunculate; inflorescence leaf with an inflated leaf-sheath; basal bract flat; cincinni alternate or fascicle-like, 1–3-flowered, pedunculate, rarely sessile, internodes elongate, rarely contracted. *Flowers* sessile, chasmogamous, tristylous, zygomorphic, non-enantiostylous, perianth connate forming a tube, hypocrateriform, spirally-coiled at post-anthesis, non-deliquescent and tightly enclosing the developing fruit, lobes 3 superior and 3 inferior, rarely 5 superior and 1 inferior, the central superior lobe with a nectar guide, consisting of 2 yellowish-green to green spots, generally surrounded by a dark purple to bluish-purple, rarely white blur; stamens dimorphic, filaments free from each other, J-shaped, glandular-pubescent, anthers dorsifixed, rimose; ovary with 3 fertile locules, multi-ovulate, septal nectaries present, style glandular-pubescent, stigma capitate to trilobate. *Capsules* loculicidal, ellipsoid to oblongoid; anthocarp thickened, ridged. *Seeds* subglobose to broadly oblongoid, testa longitudinally winged.

##### Circumscription.

Pontederiasubg.Cabanisia is composed by *P.meyeri* (A.G.Schulz) M.Pell. & C.N.Horn *comb. nov.*, *P.paniculata* Spreng. and *P.paradoxa* Mart. All three species occur in moist environments or shallow waters, being similar in habit to well-known species of P.subg.Pontederia, such as *P.cordata* L. Nonetheless, both subgenera can be differentiated based on gynoecium, fruit and seed morphology.

##### Distribution.

Mainly Central-West and Northeastern Brazil (reaching Argentina and Paraguay), growing in temporary water bodies in the Caatinga, Cerrado and Chaco domains. However, two species have very peculiar disjunctions in their distributions, also occurring in north-western South America (Colombia, Ecuador, Guyana and Venezuela), Central America (Costa Rica, Guatemala and Nicaragua), Antilles (Jamaica) and North America (Mexico).

### Key to the species of Pontederiasubg.Cabanisia

**Table:** 

1	Petiolate blades without posterior divisions, elliptic to lanceolate or narrowly ovate in outline; inflorescences 2–5-flowered, sessile, cincinni sessile, fascicle-like; perianth arranged in a 5+1 pattern, tube 2–2.5 cm long	***P.paradoxa* Mart.**
–	Petiolate blades with posterior divisions, cordate to broadly ovate in outline; inflorescences 10–many-flowered, pedunculate, cincinni pedunculate, alternate; perianth arranged in a 3+3 pattern, tube 0.8–1.6 cm long	**2**
2	Main axis with a mixture of glandular and eglandular hairs, basal bract with cordate base and caudate apex, basal cincinni 1–2(–3)-flowered, bracteoles present; central superior perianth lobe with one green spot, surrounded by purple striations, all stamens exserted from the floral tube, anthers yellow	***P.meyeri* (A.G.Schulz) M.Pell. & C.N.Horn**
–	Main axis glandular-pubescent, basal bract with round base and acute to acuminate apex, basal cincinni 4–9-flowered, bracteoles absent; central superior perianth lobe with two green spots, surrounded by a white blur, 3 stamens included and 3 stamens exserted from the floral tube, anthers bluish-lilac to lilac	***P.paniculata* Spreng.**

#### 1.1. Pontederia meyeri

Taxon classificationPlantaeCommelinalesPontederiaceae

(A.G.Schulz) M.Pell. & C.N.Horn comb. nov.

urn:lsid:ipni.org:names:60476935-2

Eichhornia meyeri A.G.Schulz, Darwiniana 6: 56. 1942. Lectotype (designated here). ARGENTINA. Chaco, Cote Lai, 25 June 1939, fl., fr., *T. Meyer 2640* (SI barcode SI000621!; isolectotypes: GH barcode GH00057534!, LIL barcode LIL000196!).

##### Distribution.

Restricted to Argentina, Paraguay and Brazil (states of Ceará, Mato Grosso and Mato Grosso do Sul).

##### Nomenclatural notes.

Schulz (1942), when describing his new *E.meyeri*, cites two specimens from the same collection, one housed at SI and another at GH. Furthermore, a third specimen, housed at LIL was found by us. After carefully analysing the syntypes, we noticed that the specimen at SI perfectly matches the original illustration. Furthermore, it is widely known that Schulz worked at the Instituto de Botánica Darwinion, thus, making the specimen at SI the obvious choice of a lectotype.

##### Taxonomical notes.

Current databases (eMonocot 2010; The Plant List 2013; Govaerts 2018; Tropicos.org 2018) have treated *E.meyeri* (≡ *P.meyeri*) as a synonym of *E.paniculata* (≡ *P.paniculata*). Nonetheless, as indicated in our identification key and by Horn (1998), both species are distinct, being easily differentiated in the field and herbaria. Thus, *E.meyeri* is here re-established and transferred to *Pontederias.l.*

#### 1.2. Pontederia paniculata

Taxon classificationPlantaeCommelinalesPontederiaceae

Spreng., Neue Entdeck. Pflanzenk. 3: 18. 1822.

Piaropus paniculatus (Spreng.) Small, Fl. S.E. U.S. (ed. 2): 1328. 1913.
Eichhornia paniculata (Spreng.) Solms, Monogr. Phan. 4: 530. 1883.
Cabanisia caracasana Klotzsch *ex* Schltdl., Abh. Naturf. Ges. Halle 6: 176. 1862, nom. superfluous. Neotype (designated here). BRAZIL. S.loc., fl., Mar 1817, M. Wied s.n. (BR barcode BR0000005188734!).

##### Distribution.

*Pontederiapaniculata* possesses a peculiarly disjunctive distribution between North-eastern Brazil (states of Alagoas, Bahia, Ceará, Paraíba, Pernambuco, Rio Grande do Norte and Sergipe), north-western South America (Colombia, Ecuador, Guyana and Venezuela), Central America (Nicaragua), Antilles (Cuba and Jamaica) and North America (Mexico).

##### Nomenclatural notes.

When describing *P.paniculata*, Sprengel (1822) makes no mention of any specimen, just mentioning that his newly described species is native to Brazil. According to Stafleu and Cowan (1985), Sprengel’s herbarium was acquired by B, but later entirely lost during the WWII. The specimen *Wied s.n.* (BR0000005188734) is an excellent match to the diagnosis provided by Sprengel, was collected prior to the publication of *P.paniculata* and was originally part of the Martius Herbarium. Despite having no proof that this specimen might have been examined by Sprengel, this specimen was surely available at the time of the publication, being originally identified as *P.paniculata* and later examined by Seubert (1847) and identified as *Eichhorniatricolor* Seub, thus making it a good choice for a neotype for *P.paniculata* and being here designated as such.

##### Taxonomical notes.

The very evident disjunctions in the distribution of *P.paniculata* might indicate a species complex, instead of a sole species. Nonetheless, we believe that without proper studies, it would be precocious to re-establish any names or recognise any new taxa at this time.

#### 1.3. Pontederia paradoxa

Taxon classificationPlantaeCommelinalesPontederiaceae

Mart. in Schultes & Schultes f., Syst. Veg. (ed. 15 bis) 7(2): 1144. 1830.

Eichhornia paradoxa (Mart.) Solms, Monogr. Phan. 4: 531 1883.
Eichhornia schultesiana Seub., Fl. Bras. 3(1): 94. 1847, nom. superfluous. Lectotype (designated here). BRAZIL. Maranhão: Alcântara oppidium at ad Porto de Carvalho, fl., fr., 1817, C.F.P. Martius 2575 (M barcode M0242209!).

##### Distribution.

*Pontederiaparadoxa* has a disjunctive distribution between Northern and North-eastern Brazil (states of Pará, Bahia, Ceará, and Rio Grande do Norte), north-western South America (Venezuela) and Central America (Costa Rica and Guatemala).

##### Nomenclatural notes.

In the original description of *P.paradoxa* (Schultes and Schultes f. 1830), it is mentioned that the description was based on a Martius collection, from the state of Maranhão, Brazil. After consulting M, we came across the specimen *Martius 2575* (M0242209) that matches the protologue in great detail. Thus, it is the obvious choice for a lectotype. Later, Seubert (1847) noticed that *P.paradoxa* did not fit in the circumscription of *Pontederia* at the time. When describing *E.schultesiana*, Seubert clearly mentions *P.paradoxa*, even citing the *Martius 2575* specimen. According to the Code (McNeill et al. 2012, Art. 52.1.), Seubert provided a superfluous replacement name, thus rendering *E.schultesiana* illegitimate.

##### Taxonomical notes.

Similarly as *P.paniculata*, *P.paradoxa* possesses a highly mind-boggling distribution, which makes us believe that it might actually represent a species complex. Two names are available for the putative disjunctive taxa, but since *P.paradoxa* in its current circumscription is known for only a handful of specimens, we discourage any taxonomic changes before the species is properly studied.

#### 2. Pontederia subg. Monochoria

Taxon classificationPlantaeCommelinalesPontederiaceae

(C.Presl) M.Pell. & C.N.Horn comb. et stat. nov.

urn:lsid:ipni.org:names:77188078-1

Fig. 6

Monochoria C.Presl, Reliq. Haenk. 1(2): 127. 1827. Type species. Monochoriahastifolia C.Presl., nom. illeg. (≡ P.hastata L.).
Calcarunia Raf., Med. Fl. 2: 106. 1830. Type species. Calcaruniahastata (L.) Raf., nom. inval. (≡ P.hastata L.).
Carigola Raf., Fl. Tellur. 2: 10. 1837. Type species. Carigolahastata (L.) Raf. (≡ P.hastata L.).
Gomphima Raf., Fl. Tellur. 2: 10. 1837. Type species. Gomphimavaginalis (Burm.f.) Raf. (≡ P.vaginalis Burm.f.).
Kadakia Raf. Fl. Tellur. 2: 9. 1837. Type species. Kadakiadilatata (Buch.-Ham.) Raf. (= P.hastata L.). **Syn. nov.**
Limnostachys F.Muell., Fragm. 1: 24. 1858. Type species. Limnostachyscyanea F.Muell. [≡ P.cyanea (F.Muell.) M.Pell. & C.N.Horn].

##### Description.

*Herbs* perennial or annual, aquatic to amphibious, erect-emergent or procumbent-emergent. *Rhizome* short to elongated. *Stems* erect, spongy, branching at the base. *Sessileleaves* early deciduous. *Petiolateleaves* distichously to spirally-alternate, congested at the apex of the stem, sometimes evenly distributed along the stem, floating to emergent, ligule truncate, petioles not-inflated, blades cordate to broadly cordate, rarely elliptic to narrowly ovate. *Main florescences (inflorescences)* terminal, sessile or pedunculate; inflorescence leaf with an inflated leaf-sheath; basal bract tubular, apex caudate, sometimes acute to acuminate, rarely leaf-like; cincinni alternate or fascicle-like, 1–3-flowered, sessile or pedunculate, internodes elongate, rarely contracted. *Flowers* pedicellate, chasmogamous, monostylous, actinomorphic or zygomorphic, enantiostylous, perianth connate only at base, campanulate, spirally-coiled at post-anthesis, non-deliquescent and tightly enclosing the developing fruit, lobes 3 superior and 3 inferior, the central superior lobe lacking a nectar guide; stamens unequal, filaments connate forming a petalo-staminal tube, J-shaped or recurved-decurved, glabrous, anthers basifixed, poricidal, dehiscent through two apical pores; ovary with 3 fertile locules, multi-ovulate, septal nectaries absent, style glabrous, stigma capitulate to capitate or trilobate to trifid. *Capsules* loculicidal, ellipsoid to oblongoid to subglobose; anthocarp thickened, ridged. *Seeds* cylindrical or ellipsoid to narrowly oblongoid to broadly oblongoid to subglobose or ovoid, testa longitudinally winged.

##### Circumscription.

Pontederiasubg.Monochoria is composed of ten exclusively Paleotropical species. All species occur in permanently moist environments or shallow waters, growing either as erect or procumbent-emergent, resembling in habit smaller members of P.subg.Pontederia and even some species of *Heteranthera*. The members of this subgenus are quite unique within *Pontederias.l.* due to their pedicellate flowers, perianth only basally connate, unequal stamens, basifixed and poricidal anthers and due to the secondary loss of the septal nectaries.

##### Distribution.

Exclusively Paleotropical (Cook 1989), with two species native to Africa (Verdcourt 1961), four to Australia (two endemic, Aston 1985) and six to Asia (Wang et al. 2004).

### Key to the species of Pontederiasubg.Monochoria

**Table:** 

1	Filaments without a tooth-like appendage, anthers yellow; stigma trilobate to trifid, with glandular hairs	**2**
–	Central inferior filament with 1(–2) tooth-like appendage, anthers greyish-blue to purple, remaining stamens with unappendaged filaments and yellow anthers; stigma capitulate to capitate, with eglandular hairs	**3**
2	Petiole of the leaves bearing inflorescences shorter than or ca. equal to the length of its leaf-sheath; anthers equal or longer than the filaments	***P.australasica* (Ridl.) M.Pell. & C.N.Horn**
–	Petiole of the leaves bearing inflorescences 2/5 to 5 times longer than its leaf-sheath; anthers smaller than the filaments	***P.cyanea* (F.Muell.) M.Pell. & C.N.Horn**
3	Basal bract leaf-like, rarely reduced to a bladeless sheath, lower cincinni 3–several-flowered; capsules ovoid; seeds cylindrical	***P.korsakowii* (Regel & Maack) M.Pell. & C.N.Horn**
–	Basal bract always reduced to a bladeless sheath, lower cincinni 1(–2)-flowered; capsules ellipsoid to broadly ellipsoid; seeds oblongoid to ellipsoid or ovoid or subglobose	**4**
4	Rhizome robust; petiolate leaves with posterior divisions with acuminate apex; flowers opening from apex to base of the inflorescence; perianth strongly spirally-coiled at post-anthesis	**5**
–	Rhizome delicate to inconspicuous; petiolate leaves with posterior divisions generally absent, if present posterior divisions with round apex; flowers opening from base to apex of the inflorescence; perianth strongly patent to slightly spirally-coiled at post-anthesis	**7**
5	Petioles longitudinally sulcate, leaves narrowly hastate or narrowly sagittate to linear sagittate, narrower than 3 cm wide; inflorescences surpassing the leaves; inner tepals obovate	***P.elata* (Ridl.) M.Pell. & C.N.Horn**
–	Petioles smooth, leaves hastate to broadly hastate or sagittate to broadly sagittate, equal or broader than 8 cm wide; inflorescences shorter than the leaves; inner tepals elliptic to oblong	**6**
6	Petiolate leaf-blades patent, posterior division 2–5 cm long; inflorescences sessile to subsessile, cincinni fascicle-like	***P.hastata* L.**
–	Petiolate leaf-blades upright, posterior division 7–11 cm long; inflorescences pedunculate, cincinni alternate	***P.valida* (G.X.Wang & Nagam.) M.Pell. & C.N.Horn**
7	Leaf blades patent; thyrsi lax, raceme- or fascicle-like, deflexed post-anthesis and in fruit; pedicels ca. as long as the floral buds	**8**
–	Leaf blades pendulous; thyrsi dense, spike-like, erect post-anthesis and in fruit; pedicels equal to shorter than ½ the length of the floral buds	**9**
8	Petiolate leaf-blades without posterior divisions, base round to obtuse, sometimes auriculate; inflorescence 2–7-flowered; seeds oblongoid, longitudinally conspicuously winged	***P.plantaginea* Roxb.**
–	Petiolate leaf-blades with conspicuous posterior divisions, base characteristically cordate; inflorescence 9–25-flowered; seeds ovoid, longitudinally inconspicuously winged	***P.vaginalis* Burm.f.**
9	Petiolateleaves cordate to ovate, leaves bearing inflorescences with petioles (5–)10–12(–16) cm long; seeds ellipsoid to narrowly oblongoid, with 8–10 longitudinal wings	***P.africana* (Solms) M.Pell. & C.N.Horn**
–	Petiolateleaves narrowly ovate to elliptic to linear, leaves bearing inflorescences with petioles (0.7–)1–2(–4) cm long; seeds subglobose to broadly oblongoid, with 12–14 longitudinal wings	***P.brevipetiolata* (Verdc.) M.Pell. & C.N.Horn**

#### 2.1. Pontederia africana

Taxon classificationPlantaeCommelinalesPontederiaceae

(Solms) M.Pell. & C.N.Horn comb. nov.

urn:lsid:ipni.org:names:77188080-1

Monochoria africana (Solms) N.E.Br., Fl. Trop. Afr. 8: 5. 1901.
Monochoria vaginalis var. africana Solms, Monogr. Phan. 4: 525. 1883. Holotype. B†; Lectotype (designated here). CENTRAL AFRICAN REPUBLIC: Djur Region, Seriba Ghattas, fl., 27 Aug 1869, G.A. Schweinfurth 2296 (PRE barcode PRE0792113-0!; isolectotypes: K barcodes K000321232!, K000321233!).

##### Distribution.

Angola, Kenya, Malawi, Mozambique, South Africa and Sudan.

##### Nomenclatural notes.

Solms-Laubach (1883) clearly designates the specimen at B as the holotype for his new taxon Monochoriavaginalisvar.africana. However, since the holotype was destroyed during WWII (Cook 1989), a lectotype is needed. The specimen at PRE is in great condition and possesses a complete preserved individual, thus being selected by us as the lectotype.

#### 2.2. Pontederia australasica

Taxon classificationPlantaeCommelinalesPontederiaceae

(Ridl.) M.Pell. & C.N.Horn comb. nov.

urn:lsid:ipni.org:names:60476936-2

Monochoria australasica Ridl., J. Straits Branch Roy. Asiat. Soc. 79: 100. 1918. Lectotype (designated by Aston 1985). AUSTRALIA. Northern Territory near Darwin, fl., fr., 4 Feb 1914, C.E.F. Allen 81 (K barcode K000873495!; isolectotype: NSW barcode NSW686319!).

##### Distribution.

Restricted to northern Australia.

#### 2.3. Pontederia brevipetiolata

Taxon classificationPlantaeCommelinalesPontederiaceae

(Verdc.) M.Pell. & C.N.Horn comb. nov.

urn:lsid:ipni.org:names:60476937-2

Monochoria brevipetiolata Verdc., Kirkia 1: 81 1961. Type. GUINEA-BISSAU. Gabú, depressões alagadas de savana entre Pitche e Canquelifá, fl., fr., 18 Sep 1950, J.V.G. Espírito Santo 2777 (holotype: K barcode K000321231!).

##### Distribution.

Gabón, Gambia, Guinea-Bissau, Ivory Coast, Mali, Níger, Senegal and Sierra Leone.

#### 2.4. Pontederia cyanea

Taxon classificationPlantaeCommelinalesPontederiaceae

(F.Muell.) M.Pell. & C.N.Horn comb. nov.

urn:lsid:ipni.org:names:60476938-2

Monochoria cyanea (F.Muell.) F.Muell., Fragm. 8: 44. 1872.
Limnostachys cyanea F.Muell., Fragm. 1: 24. 1858. Lectotype (designated by Aston 1985). AUSTRALIA. Northern Territory, Depot Creek, upper Victoria River, fl., fr., 1 Apr 1856, F.W.L. Leichhardt s.n. (K barcode K000873493!: isolectotypes: G barcode G00164431!, K barcode K000873494!, MEL barcodes MEL665251! MEL665252!).

##### Distribution.

Restricted to northern and western Australia.

#### 2.5. Pontederia elata

Taxon classificationPlantaeCommelinalesPontederiaceae

(Ridl.) M.Pell. & C.N.Horn comb. nov.

urn:lsid:ipni.org:names:77188081-1

Monochoria hastata var. elata (Ridl.) Backer, Fl. Males. 4: 258. 1951.
Monochoria elata Ridl., J. Straits Branch Roy. Asiat. Soc. 79: 99. 1918. Lectotype (designated by Cook 1989). MALAYSIA. Kedah: Jenun, fl., fr., 19 Nov 1915, M. Haniff 1208 (K barcode K000291970!; isolectotypes: BM barcode BM000958428!, K barcode K000291971!).

##### Distribution.

From Myanmar to Malaysia, Thailand and China.

##### Taxonomical notes.

*Monochoriaelata* (≡ *P.elata*) was treated by Cook (1989) as well as Guofang and Horn (2000) as an accepted name, but subsequent floras (e.g. Wang et al. 2004) and online databases (eMonocot 2010; The Plant List 2013; Govaerts 2018; Tropicos.org 2018) have either considered *M.elata* a synonym of *M.hastata* (≡ *P.hastata*) or as a variety of the latter. Nonetheless, both species can be easily differentiated based on the petiolate ornamentation, the width of the petiolate leaf-blades, length of their inflorescences and number of flowers per inflorescence. Thus, *M.elata* is here re-established and transferred to *Pontederias.l.*

#### 2.6. Pontederia hastata

Taxon classificationPlantaeCommelinalesPontederiaceae

L., Sp. Pl. 1: 288. 1753.

Monochoria hastata (L.) Solms, Monogr. Phan. 4: 523. 1883.
Carigola hastata (L.) Raf., Fl. Tellur. 2: 10. 1837.
Calcarunia hastata (L.) Raf., Med. Fl. 2: 106. 1830. Lectotype (designated by Horn and Haynes 1987). SRI LANKA. Herb. P. Hermann 2: 52, No. 129 (BM barcode BM000621681!).

##### Distribution.

Bangladesh, China, India, Indonesia, Laos, Malaysia, Myanmar, Nepal, New Guinea, Philippines, Sri Lanka, Thailand and Vietnam.

#### 2.7. Pontederia korsakowii

Taxon classificationPlantaeCommelinalesPontederiaceae

(Regel & Maack) M.Pell. & C.N.Horn comb. nov.

urn:lsid:ipni.org:names:77188082-1

Monochoria vaginalis var. korsakowii (Regel & Maack) Solms, Monogr. Phan. 4: 525. 1883.
Monochoria korsakowii Regel & Maack, Mém. Acad. Imp. Sci. Saint Pétersbourg, Sér. 7, 4(4): 155. 1861. Lectotype (designated here). RUSSIA. Ussuri, Keugxa Laa, fl., fr., 1859, R.K. Maack s.n. (LE barcode LE01007092!; isolectotypes: K barcode K000873544!; LE barcodes LE01007090!, LE01007091!, LE01007093!, P barcode P00730337!).

##### Distribution.

China, India, Indonesia, Japan, Korea, Malaysia, Pakistan, Russia, Sri Lanka and Vietnam.

##### Nomenclatural notes.

Cook (1989), in his revision for *Monochoria*, cites one of the specimens at LE as a holotype. Nonetheless, Regel and Maack (1861) make no direct mention of which herbaria the type specimens were deposited and which specimen was to be considered the type. Thus, we designate the specimen LE01007092 as the lectotype, since it possesses well-preserved flowers and seems to have been a model for the original illustration.

#### 2.8. Pontederia plantaginea

Taxon classificationPlantaeCommelinalesPontederiaceae

Roxb., Fl. Ind. (ed. 1832) 2: 123. 1832.

Monochoria vaginalis var. plantaginea (Roxb.) Solms, Monogr. Phan. 4: 524. 1883.
Monochoria plantaginea (Roxb.) Kunth, Enum. Pl. 4: 135. 1843. Lectotype (designated here). NEPAL: Nathpur, fl., Aug. 1821, N. Wallich 5096 (K barcode K001104737!; isolectotypes: K barcodes K001104733!, K001104734!, K001104735!, K001104736!, K001104738!, K001104739!, K001104740!).
Monochoria vaginalis var. angustifolia G.X.Wang, Acta Phytotax. Sin. 41: 569. 2003. Type. THAILAND. Koksung: in a marshy place, fl., 18 Sep 1984, N. Fukuoka T-36166 (holotype: KYO!; isotypes: A n.v., BKF n.v., L n.v.). **Syn. nov.**
Boottia mairei H.Lév., Cat. Pl. Yun-Nan 131. 1916. Type. CHINA. Yunnan: Dongchuan [Tangdan], fl., Aug 1912, E.E. Maire s.n. (holotype: E barcode E00386692!). **Syn. nov.**
Monochoria junghuhniana Hassk., Flora 35: 115. 1852. Lectotype (designated here). INDONESIA. Java, Yogyakarta, Djokjakarta, prope Samas ad affim Opar, fl., s.dat., Junghuhn s.n. (L barcode L0041652!). **Syn. nov.**
Monochoria linearis (Hassk.) Miq., Fl. Ned. Ind. 3: 549. 1859.
Pontederia linearis Hassk., Flora 25(2, Beibl.): 4. 1842. Type (not found). INDONESIA. Java (L?). **Syn. nov.**
Monochoria ovata Kunth, Enum. Pl. 4: 665. 1843.
Pontederia ovata Hook. & Arn., Bot. Beechey Voy. 218 1837, nom. illeg. non P.ovata L. Lectotype (designated here). SRI LANKA. Canton, fl., s.dat., Millet s.n. (G barcode G00164757!; isolectotype: E n.v.).
Pontederia cernua L. *ex* B.D.Jacks., Index Linn. Herb.: 129. 1912, nom. nud.
Pontederia alba Buch.-Ham. *ex* Wall., Numer. List: 5095 D. 1831, nom. nud.
Pontederia racemosa Buch.-Ham. *ex* Wall., Numer. List: 5095C. 1831, nom. nud.
Pontederia lanceolata Wall. *ex* Kunth, Enum. Pl. 4: 135. 1843, pro. syn.

##### Distribution.

Australia, Bangladesh, Cambodia, China, India, Indonesia, Myanmar, Nepal, Sri Lanka, Thailand and Vietnam.

##### Nomenclatural notes.

Cook (1989) cites that no suitable specimens, collected by Roxburgh, were found. Nonetheless, according to Stafleu and Cowan (1983) and Forman (1997), many of Roxburgh’s new species described after 1831 were based on specimens at the Wallich Herbarium (currently housed at K). After visiting Kew, we came across a series of specimens at Wallich Herbarium (*Wallich 5096*), collected in the Bengal region (Bangladesh, Myanmar, Nepal and India), that perfectly matched the protologue of *P.plantaginea*. One of the herbarium sheets contained several complete flowering specimens in perfect condition. Thus, we designate the gathering under the barcode K001104737, as the lectotype for *P.plantaginea*.

Hasskarl (1852), when describing *M.junghuhniana*, makes no reference to any specimens. However, the author does mention that his new species is native to Sumatra, near Samas and Opar. After analysing the collection at L, we came across a specimen from exactly the same locality as indicated in the protologue and most likely collected by Junghuhn. Thus, it is designated by us as the lectotype for *M.junghuhniana*.

##### Taxonomical notes.

*Monochoriavaginalis*, in its current circumscription (Cook 1989), is widely morphologically variable and distributed. However, recent studies (Wang et al. 2003; Tungmunnithum et al. 2016) have highlighted the need to revisit the species boundaries in this taxon. Recently, Tungmunnithum et al. (2016) published a thorough morphometric study on *M.vaginaliss.l.* from Thailand and showed that two taxa are easily recognisable. The authors informally recognised M.vaginalisBurm.f.var.vaginalis and M.vaginalisvar.angustifolia G.X.Wang as representing each of the recovered morphotypes. Nonetheless, after studying all the names treated as synonyms of *M.vaginaliss.l.* by Cook (1989), we concluded that M.vaginalisvar.angustifolia and *M.junghuhniana* are conspecific to *P.plantaginea* Roxb. Thus, *P.plantaginea* is here re-established and M.vaginalisvar.angustifolia and *M.junghuhniana* are treated as synonyms of the latter.

#### 2.9. Pontederia vaginalis

Taxon classificationPlantaeCommelinalesPontederiaceae

Burm.f., Fl. Indica: 80. 1768.

Monochoria vaginalis (Burm.f.) C.Presl *ex* Kunth, Enum. Pl. 4: 134. 1843.
Gomphima vaginalis (Burm.f.) Raf., Fl. Tellur. 2: 10. 1837.
Monochoria hastifolia C.Presl., Reliq. Haenk. 1(2): 127. 1827, nom. illeg. Lectotype (designated by Cook 1989). INDIA. Ind. Orien., fl., s.dat., W. Roxburgh s.n. (G barcode G00164756!).

##### Distribution.

Widespread throughout Asia (Afghanistan, Bangladesh, Bhutan, Cambodia, China, India, Indonesia, Iran, Japan, Korea, Laos, Malaysia, Myanmar, Nepal, Pakistan, Philippines, Russia, Sri Lanka, Thailand and Vietnam) and Oceania (Australia, Fiji, Papua New Guinea and Pacific Islands).

#### 2.10. Pontederia valida

Taxon classificationPlantaeCommelinalesPontederiaceae

(G.X.Wang & Nagam.) M.Pell. & C.N.Horn comb. nov.

urn:lsid:ipni.org:names:60476939-2

Monochoria valida G.X.Wang & Nagam., Acta Phytotax. Geobot. 45(1): 41. 1994. Type. CHINA. Hainan: Sanya, Yanglan, fl., 21 Sep 1990, G.X. Wong 901001 (holotype: WH; isotype: KYO!).

##### Distribution.

Southern China and Thailand.

##### Taxonomical notes.

*Monochoriavalida* (≡ *P.valida*) was described by Wang and Nagamasu (1994), being compared to *M.elata* (≡ *P.elata*) and *M.hastata* (≡ *P.hastata*). These species are morphologically similar, due to their robust rhizomes, petiolate blades hastate to sagittate and posterior division with acuminate apex. However, they can be easily differentiated by inflorescence morphology (inflorescence sessile, many-flowered, not surpassing the leaves and cincinni fascicle-like in *P.hastata*; inflorescence pedunculate, many-flowered, surpassing the leaves and alternate cincinni in *P.valida*; inflorescence pedunculate, few-flowered, surpassing the leaves and alternate cincinni in *P.elata*). Aside from that, leaf morphology is also helpful in species delimitation in this group. Thus, *M.valida* is here re-established and transferred to *Pontederias.l.*

#### 3. Pontederia subg. Oshunae

Taxon classificationPlantaeCommelinalesPontederiaceae

M.Pell. & C.N.Horn subg. nov.

urn:lsid:ipni.org:names:77188083-1

Fig. 7

Piaropus Raf., Fl. Tellur. 2: 81. 1837, nom. rej. Type species. Piaropusmesomelas Raf., nom. illeg. (≡ Pontederiacrassipes Mart.). **Syn. nov.**

##### Type species.

*Pontederiacrassipes* Mart. [≡ *Eichhorniacrassipes* (Mart.) Solms].

##### Description.

*Herbs* perennial, aquatic, free-floating. *Rhizome* short and inconspicuous. *Stems* inconspicuous, unbranched, producing stolons. *Sessileleaves* early deciduous. *Petiolateleaves* spirally-alternate, congested at the apex of the stem, emergent, ligule flabellate, petioles inflated, blades broadly ovate to cordate to reniform. *Main florescences (inflorescences)* terminal; inflorescence leaf without an inflated leaf-sheath; basal bract tubular; cincinni alternate, 1(–2)-flowered, sessile, internodes contracted. *Flowers* sessile, tristylous, zygomorphic, non-enantiostylous, perianth connate forming a tube, hypocrateriform, spirally-coiled at post-anthesis, deliquescent and loosely enclosing the developing fruit, 3 superior and 3 inferior, the central superior lobe with a nectar guide, consisting of 1 yellow spot, surrounded by a dark purple to bluish-purple blur; stamens dimorphic, filaments free from each other, J-shaped, glandular-pubescent, anthers dorsifixed, rimose; ovary with 3 fertile locules, multi-ovulate, septal nectaries present, style glandular-pubescent, stigma capitate to trilobate. *Capsules* loculicidal, oblongoid; anthocarp thin, smooth. *Seeds* oblongoid, testa longitudinally winged.

##### Circumscription.

Pontederiasubg.Oshunae is monospecific, being composed solely by *P.crassipes*.

##### Distribution.

Widespread throughout South America.

##### Etymology.

The name of this new subgenus derives from the Yoruba words “*Oxum*”, “*Oshun*” and “*Osun*”. These are the names given in the Candomblé religion to the orisha (i.e. a deity that reflects one of the manifestations of God) mother and guardian of freshwater bodies. Oshun is known for her beauty and vanity, being also known as the deity of luxury, pleasure, sexuality, fertility, beauty and love. The sole species accepted in Pontederiasubg.Oshunae is commonly named “*mãe d’água*” (i.e. mother of the freshwaters) in Brazil, also one of the popular names for Oshun. This popular name in Brazil makes reference to the water-hyacinth’s ability to dominate freshwater environments, as well as its ability to produce beautiful flowers.

#### 3.1. Pontederia crassipes

Taxon classificationPlantaeCommelinalesPontederiaceae

Mart., Nov. Gen. Sp. Pl. 1: 9. 1823.

Eichhornia crassipes (Mart.) Solms, Monogr. Phan. 4: 527. 1883.
Piaropus mesomelas Raf., Fl. Tellur. 2: 81. 1837, nom. illeg. Lectotype (designated by Horn 1994). BRAZIL. Bahia. Provinciae Minas Gerais, in stagnis ad fl. St. Francisci prope Malhada, s.dat., C.F.P. Martius 60 (M barcode M0242217!).

##### Distribution.

Widespread throughout South America and naturalised worldwide.

#### 4. Pontederia subg. Eichhornia

Taxon classificationPlantaeCommelinalesPontederiaceae

(Kunth) M.Pell. & C.N.Horn comb. et stat. nov.

urn:lsid:ipni.org:names:77188084-1

Fig. 8

Eichhornia Kunth, Enum. Pl. 4: 129. 1843. Type species. Eichhorniaazurea (Sw.) Kunth. (≡ P.azurea Sw.).
Leptosomus Schltdl., Abh. Naturf. Ges. Halle 6: 174. 1862. Type species. Leptosomusnatans (P.Beauv.) Schltdl. (≡ P.natans P.Beauv.).

##### Description.

*Herbs* perennial, aquatic, procumbent-emergent. *Rhizome* short and generally inconspicuous. *Stems* trailing, spongy, branched to unbranched. *Sessileleaves* late deciduous, sometimes persistent in mature plants. *Petiolateleaves* distichously-alternate, evenly distributed along stem, emergent, ligule truncate, petioles not-inflated, blades cordate to ovate or obovate to broadly obovate to rounded. *Main florescences (inflorescences)* axillary or terminal, pedunculate; inflorescence leaf without an inflated leaf-sheath; basal bract tubular; cincinni alternate, 1–3-flowered, sessile to subsessile, internodes contracted. *Flowers* sessile, chasmogamous, tristylous or pseudo-homostylous, zygomorphic, non-enantiostylous, perianth connate forming a tube, infundibuliform, revolute at post-anthesis, deliquescent and loosely enclosing the developing fruit, lobes 3 superior and 3 inferior, rarely 5 superior and 1 inferior, the central superior lobe with a nectar guide, consisting of 2 yellowish-green to green spots, generally surrounded by a dark purple to bluish-purple, rarely white blur, coiling or post-anthesis; stamens dimorphic, filaments free from each other, J-shaped, glandular-pubescent, anthers dorsifixed, rimose; ovary with 3 fertile locules, multi-ovulate, septal nectaries present, style glabrous, stigma capitate to trilobate. *Capsules* loculicidal or with irregular dehiscence, ellipsoid to oblongoid; anthocarp thin, smooth. *Seeds* subglobose to broadly oblongoid, testa longitudinally winged.

##### Circumscription.

Pontederiasubg.Eichhornia is composed of four species. All species occur in permanently or seasonal water bodies, growing as procumbent-emergent and resembling in habit some members of P.subg.Monochoria and P.subg.Pontederia. The members of this subgenus are peculiar within *Pontederias.l.* due to their late deciduous sessile leaves (sometimes persistent throughout the plant’s entire lifespan), perianth infundibuliform, revolute at post-anthesis, deliquescent and loosely enclosing the developing fruit, glandular-pubescent filaments, glabrous styles and anthocarp thin and smooth.

##### Distribution.

Mainly Neotropical, except for *P.natans*, which is restricted to continental Africa and Madagascar.

### Key to the species of Pontederiasubg.Eichhornia

**Table:** 

1	Petiolateleaves floating, blades cordate to ovate, base auriculate to cordate; inflorescences 1–4-flowered; flowers pseudo-homostylous; margins if the internal lobes of the perianth entire	**2**
–	Petiolateleaves emergent, blades obovate to broadly obovate to rounded, base cuneate; inflorescences 5–many-flowered; flowers heterostylous; margins of the internal lobes of the perianth erose to fimbriate, rarely entire	**3**
2	Inflorescences (1–)2–4-flowered; flowers 2–3.2 cm diam., perianth lilac to bluish-lilac, central superior lobe with a yellow spot, surrounded by a purple to bluish-purple blur, filaments glandular-pubescent; capsules 3-valved	***P.diversifolia* (Vahl) M.Pell. & C.N.Horn**
–	Inflorescences 1(–2)-flowered; flowers 0.7–1 cm diam., perianth purple to mauve, central superior lobe concolorous with the remaining lobes or with a dark purple blur, filaments glabrous; capsules with irregular dehiscence	***P.natans* P.Beauv.**
3	Inflorescences axillary, much exceeding the basal bract, main axis glandular-pubescent; perianth with central superior lobe with a yellow spot, filaments glandular-pubescent; seeds monomorphic	***P.azurea* Sw.**
–	Inflorescences terminal, enclosed or approximately the same size as the basal bract, main axis glabrous; perianth with central superior lobe with a dark purple to bluish-purple blur, filaments glabrous; seeds dimorphic	***P.heterosperma* (Alexander) M.Pell. & C.N.Horn**

#### 4.1 . Pontederia azurea

Taxon classificationPlantaeCommelinalesPontederiaceae

Sw., Prodr. 57. 1788.

Eichhornia azurea (Sw.) Kunth, Enum. Pl. 4: 129. 1843.
Piaropus azureus (Sw.) Raf., Fl. Tellur. 2: 81. 1837. Type. JAMAICA. s.loc., s.dat., *Brown s.n.* (holotype: S No. S-R-5196!).

##### Distribution.

Widespread in the American continent from Mexico to Uruguay.

#### 4.2. Pontederia diversifolia

Taxon classificationPlantaeCommelinalesPontederiaceae

(Vahl) M.Pell. & C.N.Horn comb. nov.

urn:lsid:ipni.org:names:77188085-1

Eichhornia diversifolia (Vahl) Urb., Symb. Antill. 4: 147. 1903.
Heteranthera diversifolia Vahl, Enum. Pl. 2: 44. 1805. Lectotype (designated here). GUIANA. s.loc., fl., s.dat., L.C. Richard s.n. (C barcode C10017422!).

##### Distribution.

Antilles (Cuba, Dominican Republic, and Puerto Rico), Central America (Belize, Costa Rica, El Salvador, Guatemala, Honduras, Nicaragua and Panama) and South America (Bolivia, Colombia, Ecuador, French Guiana, Guyana, Suriname, Venezuela and Brazil – states of Acre, Amazonas, Amapá, Pará, Rondônia, Roraima, Tocantins, Alagoas, Bahia, Ceará, Maranhão, Paraíba, Pernambuco, Piauí, Rio Grande do Norte, Sergipe, Goiás, Mato Grosso do Sul, Mato Grosso, Minas Gerais and Rio de Janeiro).

##### Nomenclatural notes.

When describing *Heterantheradiversifolia*, Vahl (1805) makes no direct mention of any analysed specimens in which he might have based the description of his new species. The author only mentions that his new species is native to Guiana and was sent to him by “Richard”. After analysing the collection at C, we came across a specimen part of Herb. Vahlian., collected by *Richard s.n.* and identified in Vahl’s handwriting as *H.diversifolia*. Thus, it is chosen by us as the lectotype.

#### 4.3. Pontederia heterosperma

Taxon classificationPlantaeCommelinalesPontederiaceae

(Alexander) M.Pell. & C.N.Horn comb. nov.

urn:lsid:ipni.org:names:77188086-1

Eichhornia heterosperma Alexander, Lloydia 2: 170. 1939. Lectotype (designated here). GUIANA. Basin of Rupununi River, Wichabai, fl., fr., 25–26 Oct 1937, A.C. Smith 2290 (NY barcode NY00247522!; isolectotypes: F barcode F0047046F!, G barcode G00168031!, GH barcode GH00255059!, K barcode K000644009!, MO barcode MO-1936311!, NY barcode NY00247521!, P barcode P00730322!, S No. S05-5985!, U barcode U0005719!, US barcode US00091644!).

##### Distribution.

Antilles (Cuba), Central America (Belize, Costa Rica, El Salvador, Guatemala, Honduras, Nicaragua and Panama) and South America (Bolivia, Colombia, Ecuador, French Guiana, Guyana and Suriname, Venezuela and Brazil – states of Acre, Amazonas, Amapá, Pará, Rondônia, Tocantins, Alagoas, Bahia, Ceará, Maranhão, Paraíba, Pernambuco, Piauí, Rio Grande do Norte, Sergipe, Goiás, Mato Grosso do Sul, Mato Grosso and Minas Gerais).

#### 4.4. Pontederia natans

Taxon classificationPlantaeCommelinalesPontederiaceae

P.Beauv., Fl. Oware 2: 18. 1807.

Eichhornia natans (P.Beauv.) Solms, Abh. Naturwiss. Vereins Bremen 7: 254. 1882.
Leptosomus natans (P.Beauv.) Schltdl., Abh. Naturf. Ges. Halle 6: 174. 1862. Lectotype (designated here). NIGERIA. Benin, fleuve Formosa, fl., fr., s.dat., A.M.F. Palisot de Beauvois s.n. (G on 3ex barcode G00418251!; isolectotype: G-DC on 4ex GDC048496!).

##### Distribution.

Angola, Benin, Botswana, Burkina Faso, Cameroon, Central African Republic, Chad, Congo, Egypt, Ethiopia, Gabon, Gambia, Ghana, Guinea, Guinea-Bissau, Ivory Coast, Liberia, Madagascar, Mali, Mozambique, Niger, Nigeria, Rwanda, Senegal, Sierra Leone, South Sudan, Sudan, Tanzania, Togo, Uganda, Zaire, Zambia and Zimbabwe.

##### Nomenclatural notes.

When describing *P.natans*, Palisot de Beauvois (1807) comments that his new species is common at the margins of the Formosa River (currently called Benin River). After analysing specimens from G and G-DC herbarium, we came across two specimens, mounted on seven sheets. The specimen GDC048496 is mounted on four sheets, composed of several flowering and fruiting specimens, with an extremely detailed annotation in the handwriting of Palisot de Beauvois. Nonetheless, the specimen G00418251 is mounted on three sheets, with the second sheet possessing a detached petiolate leaf and a copy of the original illustration and the third possessing the specimen on which the illustration was based. Thus, the G00418251 specimen is the obvious choice for a lectotype.

##### Taxonomical notes.

The African *E.natans* (≡ *P.natans*) is currently treated as a synonym of the Neotropical *Eichhorniadiversifolia* (≡ *P.diversifolia*) by all online databases (i.e. eMonocot 2010; The Plant List 2013; Govaerts 2018; Tropicos.org 2018). Nonetheless, as indicated in our identification key (see above), both species can be easily differentiated based on the number of flowers per inflorescence, floral diameter, presence or absence of a nectar guide, pubescence of the filaments and capsule dehiscence. Thus, *P.natans* is here re-established.

#### 5. Pontederia L. subg. Pontederia

Taxon classificationPlantaeCommelinalesPontederiaceae

Fig. 9

Michelia Adans., Fam. Pl. 2: 201. 1763, nom. illeg. Type species (designated here). Pontederiacordata L.
Narukila Adans., Fam. Pl. 2: 54. 1763, nom. illeg. Type species (designated here). Narukilacordata (L.) Nieuwl. (≡ P.cordata L.).
Pontederaea Kuntze, Revis. Gen. Pl. 2: 718. 1891, orth. var.
Pontederas Hoffmanns., Verz. Pfl.: 137. 1824, orth. var.
Reussia Endl., Gen. Pl.: 139. 1836. Type species (designated by Lowden 1973). Reussiatriflora Endl. *ex* Seub. [≡ P.triflora (Endl. *ex* Seub.) G.Agostini et al.].
Unisema Raf. Med. Repos. 5: 352. 1808, nom. illeg. Type species. Unisemaobtusifolia (Raf.) Raf. (≡ P.cordata L.).
Umsema Raf. Med. Repos. 5: 352 1808, orth. var.
Unisemma D.A.Godron, in Orbigny CVD, Dict. Univ. Hist. Nat.: 761. 1848, orth. var.

##### Description.

*Herbs* perennial or annual, aquatic to amphibious, erect-emergent or procumbent-emergent. *Rhizome* short and generally inconspicuous. *Stems* erect or trailing, spongy, unbranched to branching only at the base to branched. *Sessileleaves* early deciduous. *Petiolateleaves* distichously-alternate, evenly distributed along the stem or congested at the apex of the stem, emergent, ligule truncate, petioles not-inflated, blades cordate to broadly cordate, rarely elliptic to lanceolate or narrowly ovate. *Main florescences (inflorescences)* terminal, sessile or pedunculate; inflorescence leaf without an inflated leaf-sheath; basal bract flat; cincinni alternate, 1–3-flowered, sessile to shortly-pedunculate, internodes contracted. *Flowers* sessile, tristylous, zygomorphic, non-enantiostylous, perianth connate forming a tube, infundibuliform, revolute at post-anthesis, non-deliquescent and loosely enclosing the developing fruit, lobes 3 superior and 3 inferior, rarely 5 superior and 1 inferior, the central superior lobe with a nectar guide, consisting of 2 yellowish-green to green spots, generally surrounded by a dark purple to bluish-purple, rarely white blur, coiling or post-anthesis; stamens dimorphic, filaments free from each other, J-shaped, glandular-pubescent, anthers dorsifixed, rimose; ovary with 1 fertile locule, 1-ovulate, septal nectaries present, style glandular-pubescent or glabrous, stigma truncate or capitate or trilobate. *Achene* ovoid or pyriform; anthocarp hardened, ridged, ridges sinuate, toothed or echinate. *Seeds* curved narrowly ovoid or ovoid, testa smooth.

##### Circumscription.

Pontederiasubg.Pontederia is circumscribed by us to comprise eight species. Our concept of P.subg.Pontederia is equivalent to the concept of *Pontederia* adopted by Lowden (1973). Nonetheless, we accept *P.triflora* as distinct from *P.subovata* and increase the number of species in the *P.cordata* complex by the re-establishment of *P.ovalis*. The members of this subgenus are peculiar within *Pontederias.l.* due to their spike-like main florescences, ovaries 1-locular by abortion, fertile locule 1-ovulate, pendulous placentation, fruit an achene, hardened and ornate anthocarps and smooth seeds.

##### Distribution.

Exclusively Neotropical.

### Key to the species of Pontederiasubg.Pontederia

**Table:** 

1	Rhizomes absent; stems elongated, trailing; leaves evenly distributed along the stem; anthocarp echinate; seeds straight, ovoid	**2**
–	Rhizomes present, short; stems short, erect; leaves congested at the apex of the stem; anthocarp toothed or with sinuate ridges; seeds curved, narrowly ovoid	**4**
2	Petiolate leaf-blades with cordate to sagittate base; inflorescences 30–80-flowered, cincinni 2–3-flowered; flowers lilac or light to medium pink, rarely white, perianth lobes with a 3+3 arrangement	***P.rotundifolia* L.f.**
–	Petiolate leaf-blades with obtuse to cuneate base; inflorescences 2–15-flowered, cincinni 1-flowered; flowers light to medium blue, rarely white, perianth lobes with a 5+1 arrangement	**3**
3	Petiolate leaf-blades emergent, elliptic to narrowly ovate to ovate to rhomboid; inflorescences (6–)8–20-flowered	***P.subovata* (Seub.) Lowden**
–	Petiolate leaf-blades floating, linear-lanceolate to linear-elliptic to linear rhomboid; inflorescences 2–4(–5)-flowered	***P.triflora* (Endl. ex Seub.) G.Agostini et al.**
4	Petioles green, blades with a thickened midvein; inflorescences and flowers covered with light yellow hairs, flowers homostylous, central superior lobe with 1 spot, anthers dark brown to black, style equal in length with the inferior stamens	***P.parviflora* Alexander**
–	Petioles red to vinaceous to purple, rarely green, blades lacking a thickened midvein; inflorescences and flower covered with hyaline hairs, flowers tristylous, central superior lobe with 2 spots, anthers yellow or greyish-blue to purple, style either shorter or longer than the inferior stamens	**5**
5	Basal bract deflexed, main axis glabrous; central superior lobe with 2 green spots, style glandular-pubescent, stigma trilobate; anthocarp with toothed ridges	***P.cordata* L.**
–	Basal bract upright, main axis velutine or sparsely to densely villose; central superior lobe with 2 yellow spots, style glabrous, stigma truncate; anthocarp with sinuate ridges	**6**
6	Petiolate leaf-blades elliptic to narrowly ovate to ovate to broadly ovate; cincinni 2–3-flowered	***P.ovalis* Mart.**
–	Petiolate leaf-blades sagittate to broadly sagittate or hastate to broadly hastate; cincinni 4–6-flowered	***P.sagittata* C.Presl**

#### 5.1. Pontederia cordata

Taxon classificationPlantaeCommelinalesPontederiaceae

L., Sp. Pl. 1: 288. 1753.

Unisema cordata (L.) Farw., Pap. Michigan Acad. Sci. 3: 91. 1924.
Narukila cordata (L.) Nieuwl., Amer. Midl. Naturalist 3: 101. 1913. Lectotype (designated by Reveal et al. 1987). UNITED STATES. Virginia and Maryland, fl., fr., s.dat., P. Kalm s.n. (LINN barcode LINN-HL407-4).
Pontederia lancifolia Muhl., Cat. Pl. Amer. Sept.: 34. 1813.
Unisema cordata fo. lancifolia (Muhl.) Farw., Pap. Michigan Acad. Sci. 3: 92. 1924.
Narukila cordata var. lancifolia (Muhl.) Nieuwl., Repert. Spec. Nov. Regni Veg. 12: 101. 1913.
Pontederia cordata var. lancifolia (Muhl.) Torr., Fl. N. Middle United States: 343. 1824. Lectotype (designated by Lowden 1973). UNITED STATES. Carolina, fl., fr., s.dat., G.H.E. Muhlenberg 242 (PH barcode PH00033652!).

##### Distribution.

Widely distributed in North, Central and South America from Canada to Uruguay and the West Indies.

##### Taxonomical notes.

*Pontederiacordata* has always been the origin of much debate and taxonomical confusion in the genus. Most of the species currently accepted by us in *Pontederias.l.* have either been confused or compared with *P.cordata*, at some point. This can be demonstrated by how many of them have been treated either as synonyms or infraspecific taxa by different authors (Fernald 1950; Lowden 1973; Godfrey & Wooten 1979; Novelo & Lot 1994). *Pontederiacordata* is morphologically and phylogenetically related to *P.lancifolia*, with only weak differences related to leaf morphology, thus should not be recognised taxonomically. Otherwise, we believe that, based on the current phylogenetic and morphological data, *P.cordata*, *P.ovalis*, *P.parviflora* and *P.sagittata* should be treated at the species level, until further studies can properly deal with the problem.

#### 5.2. Pontederia ovalis

Taxon classificationPlantaeCommelinalesPontederiaceae

Mart. ex Schult. & Schult.f., Syst. Veg. (ed. 15 bis) 7(2): 1140. 1830.

Pontederia lanceolata f. ovalis (Mart. *ex* Schult. & Schult.f.) A.Cast., Arch. Jard. Bot. Rio de Janeiro 15: 62. 1957.
Pontederia cordata var. ovalis (Mart. *ex* Schult. & Schult.f.) Solms, Monogr. Phan. 4: 533. 1883. Lectotype (designated here). BRAZIL. s.loc., fl., s.dat., C.F.P. Martius 14 (M barcode M0242238!).

##### Distribution.

Costa Rica, Guatemala, Honduras, Bolivia, Brazil (states of Bahia, Maranhão, Paraíba, Distrito Federal, Goiás, Mato Grosso, Espírito Santo, Minas Gerais, Rio de Janeiro, São Paulo, Paraná, Santa Catarina and Rio Grande do Sul), Colombia, Paraguay and Uruguay.

##### Nomenclatural notes.

When describing *P.ovalis*, Schultes and Schultes (1830) mention that their new species is based in Martius specimens from Brazil. However, the author makes no mention in which herbarium the specimens are housed or their collectors’ numbers. While consulting the specimens at M, we came across two Martius’ specimens (i.e. *Martius 14* M0242238; *Martius 16* M0242244) that matched the protologue of *P.ovalis*. Both specimens were annotated in Martius handwriting and were probably analysed by Schultes. Since the specimen *Martius 14* (M0242238) is a more complete collection, when compared with *Martius 16* (M0242244), which is composed of two detached leaves and two inflorescences, it is selected by us as the lectotype for *P.ovalis*.

##### Taxonomical notes.

*Pontederiaovalis* has been considered by most authors and online databases as either a variety (Dubs 1998, Tropicos.org 2018) or a synonym (Schulz 1942, Tropicos.org 2018) of *P.cordata*. Nonetheless, both morphologically and phylogenetically, *P.ovalis* is much more similar to *P.sagittata*, due to its pubescent inflorescence main axis and fruits with sinuate ridges. Thus, *P.ovalis* is here re-established, being also part of the *P.cordata* species complex.

#### 5.3. Pontederia parviflora

Taxon classificationPlantaeCommelinalesPontederiaceae

Alexander, N. Amer. Fl. 19: 59. 1937.

Pontederia cordata var. parviflora (Alexander) Schery, Ann. Missouri Bot. Gard. 31: 156. 1944. Lectotype (designated here). PANAMA. Camino del Boticario, near Chapo, fl., Oct 1911, H. Pittier 4556 (NY barcode NY00260019!: isolectotypes: NY barcode NY00260020!, US barcode US00091647!).

##### Distribution.

Panama, Venezuela, Colombia and Brazil (states of Tocantins, Alagoas, Ceará, Maranhão, Paraíba, Pernambuco, Piauí, Distrito Federal, Goiás, Mato Grosso do Sul, Mato Grosso, Minas Gerais and São Paulo).

#### 5.4. Pontederia rotundifolia

Taxon classificationPlantaeCommelinalesPontederiaceae

L.f., Suppl. Pl. 192 1782.

Reussia rotundifolia (L.f.) A.Cast., Lilloa 25: 593. 1952. Lectotype (designated by Lowden 1973). SURINAM. s.loc., fl., s.dat., C.G. Dahlberg 137 (LINN barcode LINN-HL407-2!; isolectotype: S No. S09-33701!).

##### Distribution.

Mexico, Belize, Costa Rica, El Salvador, Guatemala, Honduras, Nicaragua, Panama, French Guiana, Guyana, Suriname, Venezuela, Colombia, Ecuador, Peru, Bolivia, Argentina, Paraguay, Uruguay and Brazil (states of Amazonas, Pará, Rondônia, Roraima, Tocantins, Alagoas, Bahia, Maranhão, Paraíba, Pernambuco, Distrito Federal, Goiás, Mato Grosso do Sul, Mato Grosso, Minas Gerais, Rio de Janeiro, Paraná, Rio Grande do Sul and Santa Catarina).

#### 5.5. Pontederia sagittata

Taxon classificationPlantaeCommelinalesPontederiaceae

C.Presl, Reliq. Haenk. 1(2): 116. 1827.

Pontederia cordata f. sagittata (C.Presl) Solms, Monogr. Phan. 4: 533. 1883.
Pontederia cordata var. sagittata (C.Presl) Schery, Ann. Missouri Bot. Gard. 31: 157. 1944. Holotype. MEXICO. s.loc., fl., fr., s.dat., T.P.X. Haenke s.n. (PRC barcode PRC450416!).

##### Distribution.

Mexico, Costa Rica, Guatemala, Honduras, Panama and Brazil (states of Bahia, Espírito Santo, Minas Gerais, Rio de Janeiro, São Paulo, Paraná, Rio Grande do Sul and Santa Catarina).

##### Taxonomical notes.

*Pontederiasagittata* is a poorly circumscribed taxon that is morphologically similar to *P.cordata*, due to the shape of the blade of their petiolate leaves. However, it is molecularly more closely related to *P.ovalis*, having in common the anthocarp with sinuate ridges. The disjunctive distribution of *P.sagittata* is probably related to misidentified specimens and/or the presence of cryptic species in what we currently accept as *P.sagittatas.l.* Great variation in petiolate leaf shape can be observed throughout its distribution, especially in Brazil. We believe that *P.sagittata* should be properly studied, using different approaches than traditional taxonomy, in order to solve this issue.

#### 5.6. Pontederia subovata

Taxon classificationPlantaeCommelinalesPontederiaceae

(Seub.) Lowden, Rhodora 75: 478. 1973.

Reussia subovata (Seub.) Solms, Monogr. Phan. 4: 534. 1883.
Eichhornia subovata Seub., Fl. Bras. 3(1): 91. 1847. Lectotype (designated by Lowden 1973). BRAZIL. Goiás: Provincia de Goyaz, fl., 1836–1841, G. Gardner 4022 (NY barcode NY00247524!; isolectotypes: BM, G barcodes G00168015!, G00168018!, G00168019!, K barcode K000644012!, P barcodes P00730329!, P00730589!, US barcode US00091645!).

##### Distribution.

Venezuela, Guyana, Bolivia, Argentina, Paraguay and Brazil (states of Acre, Amazonas, Amapá, Pará, Tocantins, Bahia, Piauí, Goiás, Mato Grosso, Mato Grosso do Sul, Minas Gerais, São Paulo, Paraná, Rio Grande do Sul and Santa Catarina).

#### 5.7. Pontederia triflora

Taxon classificationPlantaeCommelinalesPontederiaceae

(Endl. ex Seub.) G.Agostini et al., Ernstia 27: 9. 1984.

Reussia triflora Endl. *ex* Seub., Fl. Bras. 3(1): 96. 1847. Type (not found). BRAZIL. Pohl; Sellow (B?).

##### Distribution.

Colombia, Venezuela, Guyana, Bolivia, Argentina and Brazil (states of Roraima, Mato Grosso, Mato Grosso do Sul and Minas Gerais).

##### Nomenclatural notes.

Due to the impossibility of finding the type specimen(s?) of *Reussiatriflora* in any of the visited herbaria, we do not designate any types for this name at this point.

##### Taxonomical notes.

*Pontederiatriflora* has been greatly confused with *P.subovata*, since its original description as *R.triflora* by Seubert (1847). Both species share similar habit, leaf and floral morphology. Nonetheless, in *P.triflora*, the petiolate leaf-blades are linear-lanceolate to linear-elliptic or linear rhomboid (vs. emergent and elliptic to narrowly ovate to ovate or subrhomboid in *P.subovata*) and the inflorescences are 2–4(–5)-flowered [vs. (6–)8–20-flowered]. Thus, we reaffirm *P.triflora* as an accepted name, distinct from *P.subovata*.

## Conclusions

Pontederiaceae was one of the first families of flowering plants to be the focus of studies dealing with its phylogenetic history, based on morphological, molecular and combined data (Eckenwalder and Barrett 1986; Graham and Barrett 1995; Kohn et al. 1996; Barrett and Graham 1997; Graham et al. 1998, 2002; Ness et al. 2011). Nonetheless, until very recently (Pellegrini 2017a), the taxonomy of the family remained dogmatic and outdated, with the recognition of several non-monophyletic taxa. The arguments used as the basis for maintaining such assemblages are based especially on misunderstandings of the principles of phylogenetic systematics (Schmidt-Lebuhn 2012). According to Simpson (2006), one of the main paradigms of modern phylogenetic systematics is the proposal of classification systems that accurately reflect the evolutionary history of the studied group, being simultaneously easy to use. In order to achieve that, novel classification systems should be based on molecular phylogenetic studies, together with morphological and, whenever possible, also including less common characters (e.g. anatomy, ecology, geography, palynology, micromorphology, phytochemistry etc.; Pellegrini 2017b). Furthermore, without the inclusion of morphological characters in a phylogenetic analysis, there is no way to obtain morphological synapomorphies to support the recovered relationships and any proposed new classification (Lipscomb et al. 2003; Wiens 2004; Assis and Rieppel 2011). The implementation of these ideals on the systematics of Pontederiaceae has generated not only monophyletic genera but has considerably facilitated the taxonomy of the group. With the classification implemented here, species of Pontederiaceae are easily and unambiguously placed under two genera supported by morphological and molecular data. An infrafamilial classification for Pontederiaceae has always been of little taxonomic and systematic relevance, due to the families’ reduced size. With Pontederiaceae consisting now of only two genera, the recognition of subfamilies and tribes seems rather pointless, since each genus would be placed in its own subfamily/tribe. Thus, we do not accept any taxonomic ranks between family and genus in Pontederiaceae.

## Supplementary Material

XML Treatment for Pontederia

XML Treatment for Pontederia subg. Cabanisia

XML Treatment for Pontederia meyeri

XML Treatment for Pontederia paniculata

XML Treatment for Pontederia paradoxa

XML Treatment for Pontederia subg. Monochoria

XML Treatment for Pontederia africana

XML Treatment for Pontederia australasica

XML Treatment for Pontederia brevipetiolata

XML Treatment for Pontederia cyanea

XML Treatment for Pontederia elata

XML Treatment for Pontederia hastata

XML Treatment for Pontederia korsakowii

XML Treatment for Pontederia plantaginea

XML Treatment for Pontederia vaginalis

XML Treatment for Pontederia valida

XML Treatment for Pontederia subg. Oshunae

XML Treatment for Pontederia crassipes

XML Treatment for Pontederia subg. Eichhornia

XML Treatment for Pontederia azurea

XML Treatment for Pontederia diversifolia

XML Treatment for Pontederia heterosperma

XML Treatment for Pontederia natans

XML Treatment for Pontederia L. subg. Pontederia

XML Treatment for Pontederia cordata

XML Treatment for Pontederia ovalis

XML Treatment for Pontederia parviflora

XML Treatment for Pontederia rotundifolia

XML Treatment for Pontederia sagittata

XML Treatment for Pontederia subovata

XML Treatment for Pontederia triflora

## Supplementary materials

Supplementary material 1 List of morphological characters and coding Data type: measurement Explanation note: List of morphological characters and their respective coding, used in the morphological and total evidence analyses. This is an open access article distributed under the terms of the Creative Commons Attribution License (CC BY 4.0), which permits unrestricted use, distribution, and reproduction in any medium, provided the original author and source are credited.Marco O. O. Pellegrini, Charles N. Horn, Rafael F. Almeida

Supplementary material 2 Morphological matrix Data type: matrix Explanation note: Matrix with the 27 terminals and the first 96 characters. The characters that were not coded due to lack of data of the analyzed specimens and/or from literatures sources are coded as “?”; characters that did not apply were coded as “-”; and the polymorphic characters were coded with a “/” between each state it presented. This is an open access article distributed under the terms of the Creative Commons Attribution License (CC BY 4.0), which permits unrestricted use, distribution, and reproduction in any medium, provided the original author and source are credited.Marco O. O. Pellegrini, Charles N. Horn, Rafael F. Almeida
