# Supplementary material for: Total evidence phylogeny of Pontederiaceae (Commelinales) sheds light on the necessity of its recircumscription and synopsis of Pontederia L
Source: PhytoKeys. 2018 Aug 29;(108):25–83. doi: 10.3897/phytokeys.108.27652 (PMC6160854; doi:10.3897/phytokeys.108.27652)
Supplement: Supplementary material 1 — List of morphological characters and coding [file phytokeys-108-025-s001.doc]

**Supplement 1.** List of morphological characters and coding.

1. Life cycle, duration: perennial (0); annual (1)
2. Clonal reproduction, presence: absent (0); present (1)
3. Habitat, emergence: paludal or emergent (0); mostly to completely submersed (1); terrestrial (2)
4. Habit, base: definite (0); indefinite (1)
5. Habit, attachment to the substrate: rooted (0); free-floating (1)
6. Roots, surface: water-binding/mucilaginous (0); sand-biding (1); non-binding (2)
7. Stem, rhizome: absent (0); present (1)
8. Stem, stolon: absent (0); present (1)
9. Stem, branching: unbranched to branched just at base (0); freely branching (1)
10. Stem, elongation: elongated (0); contracted (1)
11. Stem, fibrous layer: absent (0); present (1)
12. Leaves, dimorphic: absent (0); present (1)
13. Leaves, blade: unifacial (0); late bifacial (1)
14. Leaves, ptyxis: equitant (0); conduplicate-involute, enclosing the petiole of the preceding leaf (1)
15. Leaves, vascular bundles, organization: xylem and phloem alternate or circular phloem with central xylem (0); xylem abaxial, phloem adaxial + xylem and phloem alternate (1)
16. Leaves, sheath, projection (ligule): absent (0); present (1)
17. Leaves, sheath, projection (ligule), shape: truncate (0); 2-several-parted (1); flabellate (2)
18. Leaves, sessile, phyllotaxy: distichous (0); spiral (1); equitant (2)
19. Leaves, sessile, duration: early deciduous (0); late deciduous (1); persistent (2)
20. Leaves, sessile, distribution: distributed along the stem (0); congested (1)
21. Leaves, petiolate, production: never or rarely produced (0); always produced (1)
22. Leaves, petiolate, phyllotaxy: distichous (0); spiral (1)
23. Leaves, petiolate, distribution: distributed along the stem (0); congested (1)
24. Leaves, petiolate, posture: floating (0); emersed (1)
25. Leaves, petiolate, pulvinus: absent (0); present (1)
26. Leaves, petiolate, blade, consistency: membranous (0); chartaceous to coriaceous (1)
27. Leaves, petiolate, blade, overall shape: linear to tapered (0); elliptic to ovate (1); obovate (2); cordate (3); sagittate (4); rotund (5)
28. Leaves, petiolate, blade, base, posterior divisions: absent (0); present (1)
29. Synflorescence, leaf, sheath: inflated (0); not inflated (1)
30. Inflorescence, basal bract, posture: flat (0); conduplicate (1); tubular (2)
31. Inflorescence, basal bract, apex, shape: obtuse to truncate (0); acute to acuminate to aristate (1); caudate (2)
32. Inflorescence, cincinni, number per thyrse: one (0); two to many (1)
33. Inflorescence, cincinni, peduncle, presence: absent (0); present (1)
34. Inflorescence, cincinni, peduncle, internal consistency: solid (0); fistulose (1)
35. Inflorescence, cincinni, main axis, condensation: elongate (0); contracted (1)
36. Inflorescence, cincinni, flower per cincinni, number: one or two (0); three to many (1)
37. Inflorescence, in fruit, posture: erect (0); deflexed (1)
38. Flower, self-incompatibility: self-incompatible (0); self-compatible (1)
39. Flower, pedicel, length: sessile (0); pedicellate (1)
40. Flower, symmetry: actinomorphic (0); zygomorphic (1)
41. Flower, enantiostyly: absent (0); present (1)
42. Flower, morphs: monostylous or pseudomonostylous (0); tristylous (1)
43. Flower, cleistogamy: absent (0); present (1)
44. Flower, septal nectaries: absent (0); present (1)
45. Flower, tannin cells, homogeneous: absent (0); present (1)
46. Flower, tannin cells, granular: absent (0); present (1)
47. Flower, tannin cells, fibrillar: absent (0); present (1)
48. Receptacle, aerenchyma: absent (0); present (1)
49. Perianth, aerenchyma: absent (0); sparse (1); dense (2)
50. Perianth, shape: campanulate or infundibuliform or hypocrateriform (0); tubular (1); falcate (2); flat (3)
51. Perianth, tannin cells, distribution: absent to sparse (0); moderate (1); abundant (2)
52. Perianth, tannin cells, type: homogeneous (0); granular (1); fibrillar (2)
53. Perianth, at post-anthesis: coiled (0); marcescent (1)
54. Perianth, at post-anthesis, coiling, type: spirally-coiled (0); revolute (1)
55. Perianth, at post-anthesis, coiled, enclosing the fruit: loosely (0); tightly (1)
56. Perianth, conation: basally conate (0); forming a conspicuous tube (1)
57. Perianth, color: yellow to orange to red (0); white (1); pink (2); blue to lilac to purple (3)
58. Perianth, lobes, number: 4 (0); 6 (1)
59. Perianth, lobes, arrangement: 3+3 (0); 5+1 (1)
60. Perianth, lobes, shape, between one another: all equal (0); equal in the same series (1); different in the same series (2)
61. Perianth, lobes, shape, apex: acute to acuminate (0); obtuse (1)
62. Perianth, lobes, anterior lobe, base: flat (0); folded or flanged (1)
63. Perianth, lobes, anterior lobe, nectar guide: absent (0); one spot or band (1); two spots (2); dark-colored band or blur (3)
64. Androecium, stamens, fertile, number: six (0); three or one (1)
65. Androecium, filaments, insertion: straight (0); oblique (1)
66. Androecium, filaments, conation: free (0); epipetalous (1); forming a petalo-staminal tube (2)
67. Androecium, filaments, posture: straight (0); sigmoid (1); J-shaped to recurved-decurved (2)
68. Androecium, filaments, inflation: not inflated (0); inflated (1)
69. Androecium, stamens, diversity: monomorphic (0); dimorphic (1); unequal (2)
70. Androecium, stamens, filament, appendage: absent (0); present (1)
71. Androecium, stamens, anther, insertion: dorsifixed (0); basifixed (1)
72. Androecium, stamens, anther, dehiscence: rimose (0); poricidal (1)
73. Androecium, endothecium, basal thickening: absent (0); present (1)
74. Androecium, tapetum, type: glandular (0); amoeboid (1)
75. Androecium, pollen, aperture, number: monosulcate (0); bisulcate (1)
76. Androecium, pollen, exine, ornamentation: tectate-columellate (0); non-tectate-columellate (1)
77. Gynoecium, ovary, locule fertile, number: three (0); one (1); pseudomonomerous (2)
78. Gynoecium, ovary, wall, tannin cells: absent (0); present (1)
79. Gynoecium, ovary, wall, aerenchyma: absent (0); present (1)
80. Gynoecium, ovary, septae, conation: aposeptalous (0); hemiseptalous (1); synseptalous (2)
81. Gynoecium, ovary, septae, epithelial cells: absent (0); present (1)
82. Gynoecium, ovary, septae, tannin cells: absent (0); present (1)
83. Gynoecium, ovary, placentation, type: axial (0); pendulous (1); intrusive-parietal (2); axile-parietal (3)
84. Gynoecium, ovary, placentation, flanges: unflanged (0); slightly 2-flanged (1); 2-flanged (2)
85. Gynoecium, style, posture: straight to recurved-decurved (0); J-shaped (1);
86. Gynoecium, style, pubescence: glabrous (0); pubescent or glandular-pubescent (1)
87. Gynoecium, stigma, shape: truncate (0); evenly trilobate to trifid or capitate (1); unevenly trilobate (2)
88. Gynoecium, stigma, moisture: dry (0); wet (1)
89. Fruit, type: capsule (0); achene (1)
90. Fruit, seeds per locule: one (0); several (1)
91. Fruit, anthocarp, presence: absent (0); present (1)
92. Fruit, anthocarp, envelopment of the fruit: loose (0); tight (1)
93. Fruit, anthocarp, development: thin (0); hardened (1)
94. Fruit, anthocarp, ornamentation: smooth (0); ridged (1)
95. Seed, testa, ornamentation: smooth (0); longitudinally winged or striated (1); tuberculate (2)
96. Chemistry, phenalenones: absent (0); present (1)
