# Supplementary material for: Total evidence phylogeny of Pontederiaceae (Commelinales) sheds light on the necessity of its recircumscription and synopsis of Pontederia L
Source: PhytoKeys. 2018 Aug 29;(108):25–83. doi: 10.3897/phytokeys.108.27652 (PMC6160854; doi:10.3897/phytokeys.108.27652)
Supplement: Supplementary material 2 — Morphological matrix [file phytokeys-108-025-s002.doc]

**Supplement 2.** Matrix with the 27 terminals and the first 29 characters. The characters that were not coded due to lack of data of the analyzed specimens and/or from literatures sources are coded as “?”; characters that did not apply were coded as “-”; and the polymorphic characters were coded with a “/” between each state it presented.

| **Taxon** | **1** | **2** | **3** | **4** | **5** | **6** | **7** | **8** | **9** | **10** | **11** | **12** | **13** | **14** | **15** | **16** | **17** | **18** | **19** | **20** | **21** | **22** | **23** | **24** | **25** | **26** | **27** | **28** | **29** |
| --- | --- | --- | --- | --- | --- | --- | --- | --- | --- | --- | --- | --- | --- | --- | --- | --- | --- | --- | --- | --- | --- | --- | --- | --- | --- | --- | --- | --- | --- |
| ***Helmholtzia acorifolia*** | 0 | 1 | 0 | 0 | 0 | 2 | 1 | 0 | 0 | 1 | 0 | 0 | 0 | 0 | 0 | 0 | - | 2 | 2 | 1 | 0 | - | - | - | - | - | - | - | 1 |
| ***Philydrum lanuginosum*** | 0 | 1 | 0 | 0 | 0 | 2 | 1 | 0 | 0 | 1 | 0 | 0 | 0 | 0 | 0 | 0 | - | 2 | 2 | 1 | 0 | - | - | - | - | - | - | - | 1 |
| ***Anigozanthos flavidus*** | 0 | 1 | 2 | 0 | 0 | 1 | 1 | 0 | 0 | 1 | 1 | 0 | 0 | 0 | 0 | 0 | - | 2 | 2 | 1 | 0 | - | - | - | - | - | - | - | 1 |
| ***Xiphidium caeruleum*** | 0 | 1 | 2 | 0 | 0 | 1 | 1 | 0 | 0 | 1 | 1 | 0 | 0 | 0 | 0 | 0 | - | 2 | 2 | 0 | 0 | - | - | - | - | - | - | - | 1 |
| ***Monochoria cyanea*** | 1 | 1 | 0/1 | 0 | 0 | 2 | 1 | 0 | 0 | 1 | 0 | 1 | 1 | 1 | 1 | 1 | 0 | 0 | 0 | 1 | 1 | 0 | 1 | 0/1 | 1 | 1 | 1 | 0 | 0 |
| ***Monochoria korsakovii*** | 1 | 1 | 0 | 0 | 0 | 2 | 1 | 0 | 0 | 1 | 0 | 1 | 1 | 1 | 1 | 1 | 0 | 0 | 0 | 1 | 1 | 0 | 1 | 1 | 1 | 1 | 3 | 1 | 0 |
| ***Monochoria hastata*** | 1 | 1 | 0 | 0 | 0 | 2 | 1 | 0 | 0 | 1 | 0 | 1 | 1 | 1 | 1 | 1 | 0 | 0 | 0 | 1 | 1 | 0 | 1 | 1 | 1 | 1 | 4 | 1 | 0 |
| ***Monochoria vaginalis*** | 1 | 1 | 0/1 | 0 | 0 | 2 | 1 | 0 | 0 | 1 | 0 | 1 | 1 | 1 | 1 | 1 | 0 | 0 | 0 | 1 | 1 | 0 | 1 | 0/1 | 1 | 1 | 1/3 | 1 | 0 |
| ***Eichhornia crassipes*** | 0 | 1 | 0 | 0 | 1 | 2 | 1 | 1 | 0 | 1 | 0 | 1 | 1 | 1 | 1 | 1 | 2 | 0 | 0 | 1 | 1 | 1 | 1 | 1 | 1 | 1 | 1/3 | 0/1 | 1 |
| ***Eichhornia crassipes2*** | 0 | 1 | 0 | 0 | 1 | 2 | 1 | 1 | 0 | 1 | 0 | 1 | 1 | 1 | 1 | 1 | 2 | 0 | 0 | 1 | 1 | 1 | 1 | 1 | 1 | 1 | 1/3 | 0/1 | 1 |
| ***Eichhornia diversifolia*** | 0 | 1 | 1 | 0 | 0 | 2 | 1 | 0 | 1 | 0 | 0 | 1 | 1 | 1 | 1 | 1 | 0 | 0 | 1 | 1 | 1 | 0 | 0 | 0 | 1 | 1 | 3/5 | 1 | 1 |
| ***Eichhornia azurea*** | 0 | 1 | 1 | 0 | 0 | 2 | 1 | 0 | 1 | 0 | 0 | 1 | 1 | 1 | 1 | 1 | 0 | 0 | 1 | 1 | 1 | 0 | 0 | 1 | 1 | 1 | 1/2/5 | 0 | 1 |
| ***Eichhornia heterosperma*** | 0 | 1 | 1 | 0 | 0 | 2 | 1 | 0 | 1 | 0 | 0 | 1 | 1 | 1 | 1 | 1 | 0 | 0 | 1 | 1 | 1 | 0 | 0 | 1 | 1 | 1 | 1/2/5 | 0 | 1 |
| ***Eichhornia paniculata*** | 1 | 0 | 0 | 0 | 0 | 2 | 1 | 0 | 0 | 1 | 0 | 1 | 1 | 1 | 1 | 1 | 0 | 0 | 0 | 1 | 1 | 0 | 1 | 1 | 1 | 1 | 3/4 | 1 | 0 |
| ***Eichhornia paradoxa*** | 1 | 0 | 0 | 0 | 0 | 2 | 1 | 0 | 0 | 1 | 0 | 1 | 1 | 1 | 1 | 1 | 0 | 0 | 0 | 1 | 1 | 0 | 1 | 1 | 1 | 1 | 1 | 0 | 0 |
| ***Pontederia cordata*** | 0 | 1 | 0 | 0 | 0 | 2 | 1 | 0 | 0 | 1 | 0 | 1 | 1 | 1 | 1 | 1 | 0 | 0 | 0 | 1 | 1 | 0 | 1 | 1 | 1 | 1 | 1/3 | 1 | 1 |
| ***Pontederia lancifolia*** | 0 | 1 | 0 | 0 | 0 | 2 | 1 | 0 | 0 | 1 | 0 | 1 | 1 | 1 | 1 | 1 | 0 | 0 | 0 | 1 | 1 | 0 | 1 | 1 | 1 | 1 | 0 | 0 | 1 |
| ***Pontederia rotundifolia*** | 0 | 1 | 0/1 | 0 | 0 | 2 | 1 | 0 | 1 | 0 | 0 | 1 | 1 | 1 | 1 | 1 | 0 | 0 | 0 | 1 | 1 | 0 | 0 | 1 | 1 | 1 | 3/4 | 1 | 1 |
| ***Pontederia ovalis*** | 0 | 1 | 0 | 0 | 0 | 2 | 1 | 0 | 0 | 1 | 0 | 1 | 1 | 1 | 1 | 1 | 0 | 0 | 0 | 1 | 1 | 0 | 1 | 1 | 1 | 1 | 1/3 | 0/1 | 1 |
| ***Pontederia sagittata*** | 0 | 1 | 0 | 0 | 0 | 2 | 1 | 0 | 0 | 1 | 0 | 1 | 1 | 1 | 1 | 1 | 0 | 0 | 0 | 1 | 1 | 0 | 1 | 1 | 1 | 1 | 3 | 1 | 1 |
| ***Heteranthera gardneri*** | 1 | 1 | 1 | 1 | 0 | 0 | 0 | 0 | 1 | 0 | 0 | 1 | 1 | 1 | 1 | 1 | 1 | 1 | 2 | 0 | 0 | - | - | - | - | - | - | - | 1 |
| ***Heteranthera rotundifolia*** | 1 | 0 | 1 | 1 | 0 | 0 | 0 | 0 | 1 | 0 | 0 | 1 | 1 | 1 | 1 | 1 | 1 | 1 | 1 | 0 | 1 | 1 | 1 | 0/1 | 0 | 0 | 1/5 | 0 | 1 |
| ***Heteranthera limosa*** | 1 | 0 | 1 | 1 | 0 | 0 | 0 | 0 | 1 | 0 | 0 | 1 | 1 | 1 | 1 | 1 | 1 | 1 | 1 | 0 | 1 | 1 | 1 | 0/1 | 0 | 0 | 0/1 | 0 | 1 |
| ***Heteranthera zosterifolia*** | 1 | 1 | 1 | 1 | 0 | 0 | 0 | 0 | 1 | 0 | 0 | 1 | 1 | 1 | 1 | 1 | 1 | 1 | 2 | 0 | 0 | 1 | 1 | 0 | 0 | 0 | 0 | 0 | 1 |
| ***Heteranthera seubertiana*** | 1 | 1 | 1 | 1 | 0 | 0 | 0 | 0 | 1 | 0 | 0 | 1 | 1 | 1 | 1 | 1 | 1 | 1 | 2 | 0 | 0 | 1 | 1 | 0/1 | 0 | 0 | 0 | 0 | 1 |
| ***Heteranthera oblongifolia*** | 1 | 0 | 1 | 1 | 0 | 0 | 0 | 0 | 1 | 0 | 0 | 1 | 1 | 1 | 1 | 1 | 1 | 1 | 1 | 0 | 1 | 1 | 1 | 0/1 | 0 | 0 | 0/1 | 0 | 1 |
| ***Heteranthera dubia*** | 0/1 | 1 | 1 | 1 | 0 | 0 | 0 | 0 | 1 | 0 | 0 | 1 | 1 | 1 | 1 | 1 | 1 | 1 | 2 | 0 | 0 | - | - | - | - | - | - | - | 1 |

**Continuation.** Matrix with the 60 terminals and characters 30 to 58.

| **Taxon** | **30** | **31** | **32** | **33** | **34** | **35** | **36** | **37** | **38** | **39** | **40** | **41** | **42** | **43** | **44** | **45** | **46** | **47** | **48** | **49** | **50** | **51** | **52** | **53** | **54** | **55** | **56** | **57** | **58** |
| --- | --- | --- | --- | --- | --- | --- | --- | --- | --- | --- | --- | --- | --- | --- | --- | --- | --- | --- | --- | --- | --- | --- | --- | --- | --- | --- | --- | --- | --- |
| ***Helmholtzia acorifolia*** | 1 | 1 | 1 | 0 | 0 | 1 | 0 | 1 | 1 | 1 | 1 | 0 | 0 | 1 | 1 | 1 | 1 | 0 | 0 | 0 | 2 | 0/1/2 | 1 | - | - | 0 | 1/2 | 0 | 1 |
| ***Philydrum lanuginosum*** | 1 | 1 | 1 | 0 | 0 | 0/1 | 0 | 1 | 1 | 1 | 1 | 0 | 0 | 0 | 1 | 1 | 1 | 0 | 0 | 0 | 2 | 0/1/2 | 1 | - | - | 0 | 0 | 0 | 1 |
| ***Anigozanthos flavidus*** | 1 | 1 | 1 | 0 | 0 | 1 | 0 | 0 | 1 | 1 | 0 | 0 | 0 | 1 | 1 | 1 | 1 | 0 | 0 | 2 | 0 | 0/1/2 | 1 | - | - | 1 | 0 | 1 | 1 |
| ***Xiphidium caeruleum*** | 1 | 1 | 1 | 0 | 0 | 1 | 0 | 0 | 1 | 1 | 1 | 0 | 0 | 1 | 1 | 1 | 1 | 0 | 0 | 3 | 0 | - | 1 | - | - | 0 | 0/1 | 1 | 1 |
| ***Monochoria cyanea*** | 2 | 1 | 0 | 1 | 0 | 0 | 0 | 0 | 1 | 0 | 1 | 0 | 0 | 0 | 1 | 0 | 0 | 1 | 2 | 0 | 1 | 0/1 | 0 | 0 | 1 | 0 | 3 | 1 | 2 |
| ***Monochoria korsakovii*** | 2 | 1 | 0 | 1 | 0 | 0/1 | 0 | 0 | 1 | 0 | 1 | 0 | 0 | 0 | 1 | 0 | 0 | 1 | 2 | 0 | 1 | 0 | 0 | 0 | 1 | 0 | 3 | 1 | 2 |
| ***Monochoria hastata*** | 2 | 1 | 0 | 1 | 1 | 0 | 1 | 0 | 1 | 0 | 1 | 0 | 0 | 0 | 1 | 1 | 0 | 1 | 2 | 0 | 1 | 0/1 | 0 | 0 | 1 | 0 | 3 | 1 | 2 |
| ***Monochoria vaginalis*** | 2 | 1 | 0 | 1 | 1 | 0 | 1 | 0 | 1 | 0 | 1 | 0 | 0 | 0 | 1 | 1 | 0 | 1 | 2 | 0 | 2 | 0/1 | 0 | 0 | 1 | 0 | 3 | 1 | 2 |
| ***Eichhornia crassipes*** | 1 | 1 | 0 | 0 | 1 | 0/1 | 1 | 0 | 0 | 1 | 0 | 1 | 0 | 1 | 1 | 1 | 0 | 1 | 2 | 0 | 2 | 0/1 | 0 | 1 | 0 | 1 | 1/2/3 | 1 | 1 |
| ***Eichhornia crassipes2*** | 1 | 1 | 0 | 0 | 1 | 0/1 | 1 | 0 | 0 | 1 | 0 | 1 | 0 | 1 | 1 | 1 | 0 | 1 | 2 | 0 | 2 | 0/1 | 0 | 1 | 0 | 1 | 1/2/3 | 1 | 1 |
| ***Eichhornia diversifolia*** | 1 | 1 | 0 | 0 | 1 | 0 | 1 | 1 | 0 | 1 | 0 | 0 | 0 | 0 | 1 | 0 | 1 | 1 | 2 | 0 | 2 | 0/2 | 0 | 1 | 0 | 1 | 2/3 | 1 | 1 |
| ***Eichhornia azurea*** | 1 | 1 | 0 | 0 | 1 | 0/1 | 1 | 1 | 0 | 1 | 0 | 1 | 0 | 1 | 1 | 0 | 1 | 1 | 2 | 0 | 1 | 2 | 0 | 1 | 0 | 1 | 1/3 | 1 | 1 |
| ***Eichhornia heterosperma*** | 1 | 1 | 0 | 0 | 1 | 0/1 | 1 | 1 | 0 | 1 | 0 | 1 | 0 | 1 | 1 | 0 | 1 | 1 | 2 | 0 | 1 | 2 | 0 | 1 | 0 | 1 | 1/3 | 1 | 1 |
| ***Eichhornia paniculata*** | 2 | 1 | 1 | 1 | 0 | 0/1 | 0 | 0 | 0 | 1 | 0 | 1 | 0 | 1 | 0 | 1 | 0 | 1 | 2 | 0 | 1 | 1 | 0 | 0 | 1 | 1 | 2/3 | 1 | 2 |
| ***Eichhornia paradoxa*** | 2 | 1 | 0 | 1 | 1 | 0 | 0 | 0 | 0 | 1 | 0 | 1 | 0 | 1 | 0 | 1 | 0 | 1 | 2 | 0 | 1 | 1 | 0 | 0 | 1 | 1 | 1/3 | 1 | 2 |
| ***Pontederia cordata*** | 1 | 1 | 0 | 0 | 1 | 0/1 | 1 | 1 | 0 | 1 | 0 | 1 | 0 | 1 | 1 | 1 | 0 | 1 | 2 | 0 | 2 | 0/1 | 0 | 1 | 1 | 1 | 1/2/3 | 1 | 1 |
| ***Pontederia lancifolia*** | 1 | 1 | 0 | 0 | 1 | 0/1 | 1 | 1 | 0 | 1 | 0 | 1 | 0 | 1 | 1 | 1 | 0 | 1 | 2 | 0 | 2 | 0/1 | 0 | 1 | 1 | 1 | 1/2/3 | 1 | 1 |
| ***Pontederia rotundifolia*** | 1 | 1 | 0 | 0 | 1 | 0 | 1 | 1 | 0 | 1 | 0 | 1 | 0 | 1 | 1 | 0 | 0 | 1 | 2 | 0 | 2 | 0 | 0 | 1 | 1 | 1 | 1/2 | 1 | 1 |
| ***Pontederia ovalis*** | 1 | 1 | 0 | 0 | 1 | 0/1 | 1 | 1 | 0 | 1 | 0 | 1 | 0 | 1 | 1 | 0 | 1 | 1 | 2 | 0 | 2 | 0/1/2 | 0 | 1 | 1 | 1 | 1/3 | 1 | 1 |
| ***Pontederia sagittata*** | 1 | 1 | 0 | 0 | 1 | 1 | 1 | 1 | 0 | 1 | 0 | 1 | 0 | 1 | 1 | 0 | 1 | 1 | 2 | 0 | 2 | 0/1/2 | 0 | 1 | 1 | 1 | 1/3 | 1 | 1 |
| ***Heteranthera gardneri*** | 0 | 0 | 1 | 0 | 1 | 0 | 1 | 0 | 0 | 1 | 0 | 0 | 1 | 0 | 1 | 0 | 0 | 1 | 1 | 1 | 1 | 0/1 | 1 | - | - | 1 | 0 | 1 | 0 |
| ***Heteranthera rotundifolia*** | 1 | 0 | 1 | 0 | 0 | 0 | 1 | 0 | 0 | 1 | 1 | 0 | 0 | 0 | 1 | 0 | 0 | 1 | 1 | 1 | 0 | - | 1 | - | - | 1 | 1/3 | 1 | 1 |
| ***Heteranthera limosa*** | 1 | 0 | 1 | 0 | 0 | 0 | 1 | 0 | 0 | 1 | 1 | 0 | 0 | 0 | 1 | 0 | 0 | 1 | 1 | 1 | 0 | - | 1 | - | - | 1 | 1/3 | 1 | 1 |
| ***Heteranthera zosterifolia*** | 0 | 0 | 1 | 0 | 0 | 0 | 1 | 0 | 0 | 1 | 1 | 0 | 1 | 0 | 1 | 0 | 0 | 1 | 1 | 1 | 0 | 0 | 1 | - | - | 1 | 3 | 1 | 0 |
| ***Heteranthera seubertiana*** | 1 | 0 | 1 | 0 | 0 | 1 | 1 | 0 | 0 | 1 | 1 | 0 | 1 | 0 | 1 | 0 | 0 | 1 | 1 | 1 | 0 | 0 | 1 | - | - | 1 | 0/1/3 | 1 | 1 |
| ***Heteranthera oblongifolia*** | 0 | 0 | 1 | 0 | 0 | 0 | 1 | 0 | 0 | 1 | 1 | 0 | 0 | 0 | 1 | 0 | 0 | 1 | 1 | 1 | 0 | 0 | 1 | - | - | 1 | 1/3 | 1 | 0 |
| ***Heteranthera dubia*** | 1 | 0 | 1 | 0 | 1 | 0 | 1 | 0 | 0 | 0 | 0 | 0 | 1 | 0 | 1 | 0 | 0 | 1 | 1 | 1 | 2 | 0/1 | 1 | - | - | 1 | 0 | 1 | 1 |

**Continuation.** Matrix with the 60 terminals and characters 59 to 87.

| **Taxon** | **59** | **60** | **61** | **62** | **63** | **64** | **65** | **66** | **67** | **68** | **69** | **70** | **71** | **72** | **73** | **74** | **75** | **76** | **77** | **78** | **79** | **80** | **81** | **82** | **83** | **84** | **85** | **86** | **87** |
| --- | --- | --- | --- | --- | --- | --- | --- | --- | --- | --- | --- | --- | --- | --- | --- | --- | --- | --- | --- | --- | --- | --- | --- | --- | --- | --- | --- | --- | --- |
| ***Helmholtzia acorifolia*** | 1 | 2 | 0 | 0 | 0 | 1 | 0 | 0 | 0 | 0 | 0 | 0 | 1 | 0 | 0 | 0 | 0 | 0 | 0 | 1 | 0 | 2 | 0 | 1 | 0 | 2 | 0 | 0 | 1 |
| ***Philydrum lanuginosum*** | 1 | 2 | 0 | 0 | 0 | 1 | 0 | 0 | 0 | 0 | 0 | 0 | 1 | 0 | 0 | 0 | 0 | 0 | 1 | 1 | 0 | 0 | 0 | 1 | 2 | 2 | 0 | 0 | 1 |
| ***Anigozanthos flavidus*** | 0 | 0 | 0 | 0 | 0 | 0 | 0 | 1 | 0 | 0 | 0 | 0 | 1 | 0 | 1 | 1 | 0 | 1 | 0 | 1 | 0 | 2 | 0 | 1 | 0 | 0 | 0 | 0 | 0 |
| ***Xiphidium caeruleum*** | 0 | 0 | 0 | 0 | 0 | 1 | 0 | 0 | 0 | 0 | 1 | 0 | 1 | 1 | 1 | 1 | 0 | 1 | 0 | 1 | 0 | 2 | 0 | 1 | 0 | 0 | 0 | 0 | 0 |
| ***Monochoria cyanea*** | 0 | 1 | 1 | 0 | 0 | 0 | 0 | 2 | 2 | 0 | 2 | 0 | 1 | 1 | 1 | 1 | 1 | 1 | 0 | 0 | 1 | 2 | 1 | 0 | 0 | 2 | 1 | 0 | 1 |
| ***Monochoria korsakovii*** | 0 | 1 | 1 | 0 | 0 | 0 | 0 | 2 | 2 | 0 | 2 | 1 | 1 | 1 | 1 | 1 | 1 | 1 | 0 | 0 | 1 | 2 | 1 | 0 | 0 | 2 | 1 | 0 | 1 |
| ***Monochoria hastata*** | 0 | 1 | 1 | 0 | 0 | 0 | 0 | 2 | 2 | 0 | 2 | 1 | 1 | 1 | 1 | 1 | 1 | 1 | 0 | 0 | 1 | 2 | 1 | 0 | 0 | 2 | 1 | 0 | 1 |
| ***Monochoria vaginalis*** | 0 | 1 | 1 | 0 | 0 | 0 | 0 | 2 | 2 | 0 | 2 | 1 | 1 | 1 | 1 | 1 | 1 | 1 | 0 | 0 | 1 | 2 | 1 | 0 | 0 | 2 | 1 | 0 | 1 |
| ***Eichhornia crassipes*** | 0 | 1 | 1 | 0 | 1 | 0 | 0 | 1 | 2 | 0 | 1 | 0 | 0 | 0 | 1 | 1 | 1 | 1 | 0 | 1 | 1 | 2 | 0 | 1 | 0 | 2 | 1 | 1 | 1 |
| ***Eichhornia crassipes2*** | 0 | 1 | 1 | 0 | 1 | 0 | 0 | 1 | 2 | 0 | 1 | 0 | 0 | 0 | 1 | 1 | 1 | 1 | 0 | 1 | 1 | 2 | 0 | 1 | 0 | 2 | 1 | 1 | 1 |
| ***Eichhornia diversifolia*** | 0 | 1 | 1 | 0 | 1 | 0 | 0 | 1 | 2 | 0 | 1 | 0 | 0 | 0 | 1 | 1 | 1 | 1 | 0 | 1 | 0 | 2 | 0 | 1 | 0 | 2 | 1 | 0 | 1 |
| ***Eichhornia azurea*** | 0 | 1 | 1 | 0 | 1 | 0 | 0 | 1 | 2 | 0 | 1 | 0 | 0 | 0 | 1 | 1 | 1 | 1 | 0 | 1 | 0 | 2 | 0 | 1 | 0 | 2 | 1 | 0 | 1 |
| ***Eichhornia heterosperma*** | 0 | 1 | 1 | 0 | 0/3 | 0 | 0 | 1 | 2 | 0 | 1 | 0 | 0 | 0 | 1 | 1 | 1 | 1 | 0 | 1 | 0 | 2 | 0 | 1 | 0 | 2 | 1 | 0 | 1 |
| ***Eichhornia paniculata*** | 0 | 1 | 1 | 0 | 2 | 0 | 0 | 1 | 2 | 0 | 1 | 0 | 0 | 0 | 1 | 1 | 1 | 1 | 0 | 0 | 1 | 1 | 0 | 0 | 0 | 2 | 1 | 1 | 1 |
| ***Eichhornia paradoxa*** | 1 | 1 | 1 | 0 | 2 | 0 | 0 | 1 | 2 | 0 | 1 | 0 | 0 | 0 | 1 | 1 | 1 | 1 | 0 | 0 | 1 | 1 | 0 | 0 | 0 | 2 | 1 | 1 | 1 |
| ***Pontederia cordata*** | 0 | 1 | 1 | 0 | 2 | 0 | 0 | 1 | 2 | 0 | 1 | 0 | 0 | 0 | 1 | 1 | 1 | 1 | 2 | 1 | 1 | 2 | 1 | 1 | 1 | 0 | 1 | 1 | 1 |
| ***Pontederia lancifolia*** | 0 | 1 | 1 | 0 | 2 | 0 | 0 | 1 | 2 | 0 | 1 | 0 | 0 | 0 | 1 | 1 | 1 | 1 | 2 | 1 | 1 | 2 | 1 | 1 | 1 | 0 | 1 | 1 | 1 |
| ***Pontederia rotundifolia*** | 1 | 1 | 1 | 0 | 2 | 0 | 0 | 1 | 2 | 0 | 1 | 0 | 0 | 0 | 1 | 1 | 1 | 1 | 2 | 1 | 1 | 2 | 1 | 1 | 1 | 0 | 1 | 1 | 1 |
| ***Pontederia ovalis*** | 0 | 1 | 1 | 0 | 2 | 0 | 0 | 1 | 2 | 0 | 1 | 0 | 0 | 0 | 1 | 1 | 1 | 1 | 2 | 1 | 1 | 2 | 1 | 1 | 1 | 0 | 1 | 1 | 1 |
| ***Pontederia sagittata*** | 0 | 1 | 1 | 0 | 2 | 0 | 0 | 1 | 2 | 0 | 1 | 0 | 0 | 0 | 1 | 1 | 1 | 1 | 2 | 1 | 1 | 2 | 1 | 1 | 1 | 0 | 1 | 1 | 1 |
| ***Heteranthera gardneri*** | 0 | 2 | 0 | 0 | 0 | 1 | 1 | 1 | 0 | 0/1 | 1 | 0 | 1 | 0 | 1 | 1 | 1 | 1 | 1 | 0 | 1 | 0 | 0 | 0 | 2 | 1 | 0 | 1 | 2 |
| ***Heteranthera rotundifolia*** | 0 | 2 | 0 | 1 | 1 | 1 | 1 | 1 | 1 | 0 | 1 | 0 | 1 | 0 | 1 | 1 | 1 | 1 | 0 | 0 | 1 | 1 | 0 | 0 | 3 | 2 | 0 | 0 | 2 |
| ***Heteranthera limosa*** | 0 | 2 | 0 | 1 | 1 | 1 | 1 | 1 | 1 | 0 | 1 | 0 | 1 | 0 | 1 | 1 | 1 | 1 | 0 | 0 | 1 | 1 | 0 | 0 | 3 | 2 | 0 | 0 | 2 |
| ***Heteranthera zosterifolia*** | 1 | 2 | 1 | 0 | 0/3 | 1 | 1 | 1 | 0 | 1 | 1 | 0 | 1 | 0 | 1 | 1 | 1 | 1 | 1 | 0 | 1 | 0 | 0 | 0 | 2 | 1 | 0 | 1 | 2 |
| ***Heteranthera seubertiana*** | 1 | 2 | 1 | 0 | 3 | 1 | 1 | 1 | 0 | 1 | 1 | 0 | 1 | 0 | 1 | 1 | 1 | 1 | 1 | 0 | 1 | 0 | 0 | 0 | 2 | 1 | 0 | 1 | 2 |
| ***Heteranthera oblongifolia*** | 0 | 2 | 0 | 1 | 1 | 1 | 1 | 1 | 1 | 0 | 1 | 0 | 1 | 0 | 1 | 1 | 1 | 1 | 0 | 0 | 1 | 1 | 0 | 0 | 3 | 2 | 0 | 1 | 2 |
| ***Heteranthera dubia*** | 0 | 0 | 0 | 0 | 0 | 1 | 1 | 1 | 0 | 1 | 1 | 0 | 1 | 0 | 1 | 1 | 1 | 1 | 1 | 1 | 1 | 0 | 0 | 1 | 2 | 1 | 0 | 0 | 2 |

**Continuation.** Matrix with the 60 terminals and characters 88 to 96.

| **Taxon** | **88** | **89** | **90** | **91** | **92** | **93** | **94** | **95** | **96** |
| --- | --- | --- | --- | --- | --- | --- | --- | --- | --- |
| ***Helmholtzia acorifolia*** | 0 | 0 | 1 | 0 | - | - | - | 1 | 0 |
| ***Philydrum lanuginosum*** | 0 | 0 | 1 | 0 | - | - | - | 1 | 0 |
| ***Anigozanthos flavidus*** | 0 | 0 | 1 | 0 | - | - | - | 1 | 1 |
| ***Xiphidium caeruleum*** | 0 | 0 | 1 | 0 | - | - | - | 2 | 1 |
| ***Monochoria cyanea*** | 1 | 0 | 1 | 1 | 1 | 1 | 1 | 1 | 0 |
| ***Monochoria korsakovii*** | 1 | 0 | 1 | 1 | 1 | 1 | 1 | 1 | 0 |
| ***Monochoria hastata*** | 1 | 0 | 1 | 1 | 1 | 1 | 1 | 1 | 0 |
| ***Monochoria vaginalis*** | 1 | 0 | 1 | 1 | 1 | 1 | 1 | 1 | 0 |
| ***Eichhornia crassipes*** | 1 | 0 | 1 | 1 | 0 | 0 | 0 | 1 | 0 |
| ***Eichhornia crassipes2*** | 1 | 0 | 1 | 1 | 0 | 0 | 0 | 1 | 0 |
| ***Eichhornia diversifolia*** | 1 | 0 | 1 | 1 | 0 | 0 | 0 | 1 | 0 |
| ***Eichhornia azurea*** | 1 | 0 | 1 | 1 | 0 | 0 | 0 | 1 | 0 |
| ***Eichhornia heterosperma*** | 1 | 0 | 1 | 1 | 0 | 0 | 0 | 1 | 0 |
| ***Eichhornia paniculata*** | 1 | 0 | 1 | 1 | 1 | 1 | 1 | 1 | 0 |
| ***Eichhornia paradoxa*** | 1 | 0 | 1 | 1 | 1 | 1 | 1 | 1 | 0 |
| ***Pontederia cordata*** | 1 | 1 | 0 | 1 | 1 | 1 | 1 | 0 | 0 |
| ***Pontederia lancifolia*** | 1 | 1 | 0 | 1 | 1 | 1 | 1 | 0 | 0 |
| ***Pontederia rotundifolia*** | 1 | 1 | 0 | 1 | 1 | 1 | 1 | 0 | 0 |
| ***Pontederia ovalis*** | 1 | 1 | 0 | 1 | 1 | 1 | 1 | 0 | 0 |
| ***Pontederia sagittata*** | 1 | 1 | 0 | 1 | 1 | 1 | 1 | 0 | 0 |
| ***Heteranthera gardneri*** | 0 | 0 | 1 | 1 | 0 | 0 | 0 | 1 | 0 |
| ***Heteranthera rotundifolia*** | 0 | 0 | 1 | 1 | 0 | 0 | 0 | 1 | 0 |
| ***Heteranthera limosa*** | 0 | 0 | 1 | 1 | 0 | 0 | 0 | 1 | 0 |
| ***Heteranthera zosterifolia*** | 0 | 0 | 1 | 1 | 0 | 0 | 0 | 1 | 0 |
| ***Heteranthera seubertiana*** | 0 | 0 | 1 | 1 | 0 | 0 | 0 | 1 | 0 |
| ***Heteranthera oblongifolia*** | 0 | 0 | 1 | 1 | 0 | 0 | 0 | 1 | 0 |
| ***Heteranthera dubia*** | 0 | 0 | 1 | 1 | 0 | 0 | 0 | 1 | 0 |
